# De novo design of proteins housing excitonically coupled chlorophyll special pairs

**DOI:** 10.1038/s41589-024-01626-0

**Published:** 2024-06-03

**Authors:** Nathan M. Ennist, Shunzhi Wang, Madison A. Kennedy, Mariano Curti, George A. Sutherland, Cvetelin Vasilev, Rachel L. Redler, Valentin Maffeis, Saeed Shareef, Anthony V. Sica, Ash Sueh Hua, Arundhati P. Deshmukh, Adam P. Moyer, Derrick R. Hicks, Avi Z. Swartz, Ralph A. Cacho, Naia Novy, Asim K. Bera, Alex Kang, Banumathi Sankaran, Matthew P. Johnson, Amala Phadkule, Mike Reppert, Damian Ekiert, Gira Bhabha, Lance Stewart, Justin R. Caram, Barry L. Stoddard, Elisabet Romero, C. Neil Hunter, David Baker

**Affiliations:** 1 Institute for Protein Design, University of Washington, Seattle, WA USA; 2 Department of Biochemistry, University of Washington, Seattle, WA USA; 3 Division of Basic Sciences, Fred Hutchinson Cancer Center, Seattle, WA USA; 4 Institute of Chemical Research of Catalonia (ICIQ-CERCA), Barcelona Institute of Science and Technology (BIST), Tarragona, Spain; 5 School of Biosciences, University of Sheffield, Sheffield, UK; 6 Department of Cell Biology and Skirball Institute of Biomolecular Medicine, New York University School of Medicine, New York, NY USA; 7 Departament de Química Física i Inorgànica, Universitat Rovira i Virgili, Tarragona, Spain; 8 Department of Chemistry and Biochemistry, University of California, Los Angeles, Los Angeles, CA USA; 9 Molecular Biophysics and Integrated Bioimaging, Berkeley Center for Structural Biology, Lawrence Berkeley National Laboratory, Berkeley, CA USA; 10 Department of Chemistry, Purdue University, West Lafayette, IN USA; 11 Department of Microbiology, New York University School of Medicine, New York, NY USA; 12 Howard Hughes Medical Institute, University of Washington, Seattle, WA USA

**Keywords:** Fluorescence resonance energy transfer, Protein design, Synthetic biology, Photosynthesis, Protein design

## Abstract

Natural photosystems couple light harvesting to charge separation using a ‘special pair’ of chlorophyll molecules that accepts excitation energy from the antenna and initiates an electron-transfer cascade. To investigate the photophysics of special pairs independently of the complexities of native photosynthetic proteins, and as a first step toward creating synthetic photosystems for new energy conversion technologies, we designed *C*_2_-symmetric proteins that hold two chlorophyll molecules in closely juxtaposed arrangements. X-ray crystallography confirmed that one designed protein binds two chlorophylls in the same orientation as native special pairs, whereas a second designed protein positions them in a previously unseen geometry. Spectroscopy revealed that the chlorophylls are excitonically coupled, and fluorescence lifetime imaging demonstrated energy transfer. The cryo-electron microscopy structure of a designed 24-chlorophyll octahedral nanocage with a special pair on each edge closely matched the design model. The results suggest that the de novo design of artificial photosynthetic systems is within reach of current computational methods.

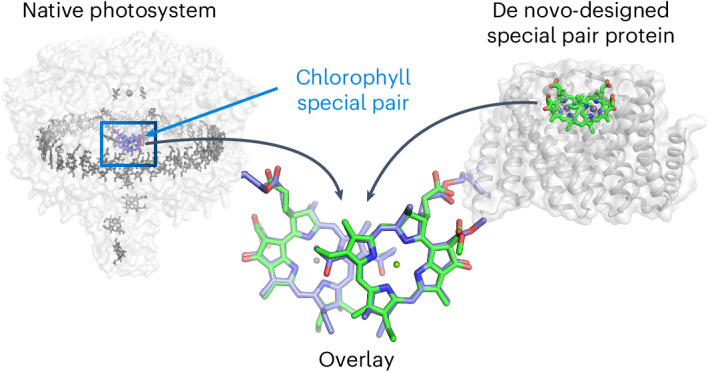

## Abstract (web summary)

A de novo-designed protein that precisely assembles a chlorophyll dimer has been developed. The design matches the conformation of the native ‘special pair’ of chlorophylls that functions as the primary electron donor in natural photosynthetic reaction centers. In the designed protein, excitonically coupled chlorophylls participate in energy transfer. The proteins were also redesigned to assemble into 24-chlorophyll nanocages.

## Main

Photosynthetic proteins manipulate the distances and angles between chlorophyll (Chl) molecules to tune excitonic coupling and control absorption and fluorescence spectra, excited state dynamics, energy transfer and electron tunneling^1–6^. This control enables light harvesting and charge separation with quantum yields of 97% or higher under favorable conditions^7,8^. Natural photosynthesis can guide the development of synthetic biology for renewable fuel production, but only if we can determine the structure−function relationships required for efficient solar-to-fuel energy conversion and build new structures that exploit this knowledge. Chl special pairs have attracted great interest as primary electron donors, but the complexity of natural photosystems makes it difficult to study these Chl molecules directly. Model protein systems, such as the water-soluble Chl protein and the B820 dimer of LH1, allow the investigation of excitonic interactions between Chl molecules or between bacteriochlorophyll (BChl) molecules without spectral congestion from other pigments^9–12^; however, the BChl and Chl dimers are not structural mimics of special pairs. The challenges of studying special pairs have inspired chemists to synthesize numerous small molecule mimics^13–17^, which have provided valuable insights, but these can be labor-intensive to synthesize, overlook the role of protein matrix effects, which are important in native special pairs^5^, and lack the fine control over Chl−Chl distances and orientations needed to reproduce the precise geometries of native special pairs. De novo-designed Zn-tetrapyrrole monomer-binding proteins^18–26^ and de novo Chl dimer proteins^27–30^ have contributed to the understanding of light harvesting and quenching of excitation energy, but no Chl dimer structures have been determined experimentally in designed proteins. Systematic methods for assembling Chl dimers with predefined geometries are lacking, making it difficult to correlate structure and function, and despite decades of active research, there has been no generalizable strategy for assembling Chl dimers that precisely match special pair geometries.

**Fig. 1:**
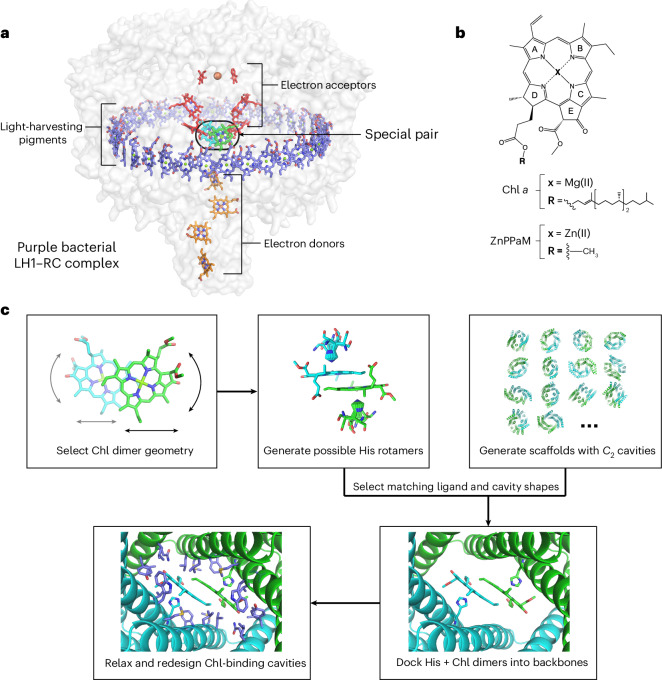
Computational design of Chl special pair proteins. **a**, Cryo-EM structure of the native photosynthetic LH1−RC complex from purple nonsulfur bacteria (*Blastochloris viridis*) (PDB 6ET5)^67^. The special pair is shown in green and cyan, electron acceptors in red, electron donors in orange and light-harvesting pigments in blue. Hydrocarbon tail groups and carotenoids have been removed for clarity. **b**, Chemical structures of two chlorin compounds, Chl *a* and ZnPPaM. Chl *a* and ZnPPaM have similar spectroscopic properties. **c**, Computational design of Chl special pair proteins begins with selection of a Chl dimer geometry and generation of inverse His rotamers. His−Chl dimers were docked into designed homodimers and the Chl-binding pockets were redesigned using Rosetta FastDesign.

We reasoned that recent advances in computational protein design could enable the creation of stable, water-soluble proteins that assemble Chl dimers with predefined geometries. Binding a small molecule as a dimer is a computational challenge because the binding interface involves not only the protein but also the second small molecule, which has an independent set of rotational and translational degrees of freedom. To control these degrees of freedom, we sought to design homodimers with perfect two-fold cyclic (*C*_2_) symmetry, which bind a *C*_2_-symmetric Chl pair such that the *C*_2_ symmetry axes of the protein and chromophore are coincident, similar to native reaction centers, which can have true *C*_2_ symmetry^31,32^ or pseudo-*C*_2_ symmetry (Fig. 1). *C*_2_ symmetry ensures that the two bound Chl molecules will have near-degenerate site energies, improving the resonance between pigment transitions necessary to create delocalized states^33^. For Chl dimer protein scaffolds, we chose hyperstable *C*_2_-symmetric repeat protein dimers containing symmetric pockets with tunable sizes and geometries^34–36^. In this dimeric repeat protein architecture (Fig. 1c), the hydrophobic core is independent of the small molecule-binding site, enabling full customization for binding with little impact on the overall protein structure. Several thousand *C*_2_-symmetric homodimers that sample a wide range of superhelical curvature, rise and radius parameters have been generated^34,35^.

## Results

### De novo protein design strategy

To investigate the effect of geometry on Chl−Chl coupling, we set out to design a range of *C*_2_-symmetric dimers that hold two closely interacting Chl molecules in varied geometries, including the arrangement found in native special pairs. In native proteins, BChl or Chl molecules typically have a pentacoordinate central Mg(II) or Zn(II) ion with a His Nε atom as the axial ligand. For each chosen special pair geometry, we built a His rotamer interaction field and stored the possible His−Chl interaction geometries in a hash table (Fig. 1c; see Methods for details). For each geometrically compatible *C*_2_ scaffold, we cycled through His−Chl rotamers from the hash table, aligned them to the scaffold *C*_2_-symmetry axis and searched for matches of the His N−Cα−C backbone atoms with the backbone atoms of the residues lining the binding cavity. Scaffolds in which the His N–Cα−C backbone atoms aligned with the corresponding atoms in the protein backbone and could also accommodate the Chl dimer without clashes were redesigned using symmetric Rosetta FastDesign to optimize hydrophobic packing and hydrogen bonding around the Chl molecules^37^ (Fig. 1c). Designs were filtered on the basis of the Rosetta full-atom energy, the solvent-accessible surface area of the Chl dimer, His rotamers and His Nε−metal ligation geometry. We selected 43 designs based on 13 unique scaffolds for experimental characterization (see Supplementary Table 1 for amino acid sequences). We also characterized an additional five redesigned variants of one of the initial 43 designs after determination of its X-ray crystal structure provided clues to improve its function (vide infra). The protein monomer sizes ranged from 20.6 to 28.4 kDa (179 to 261 amino acids). We refer to these 48 designs as Chl special pair proteins.

### Special pair protein stability and pigment binding

**Fig. 2:**
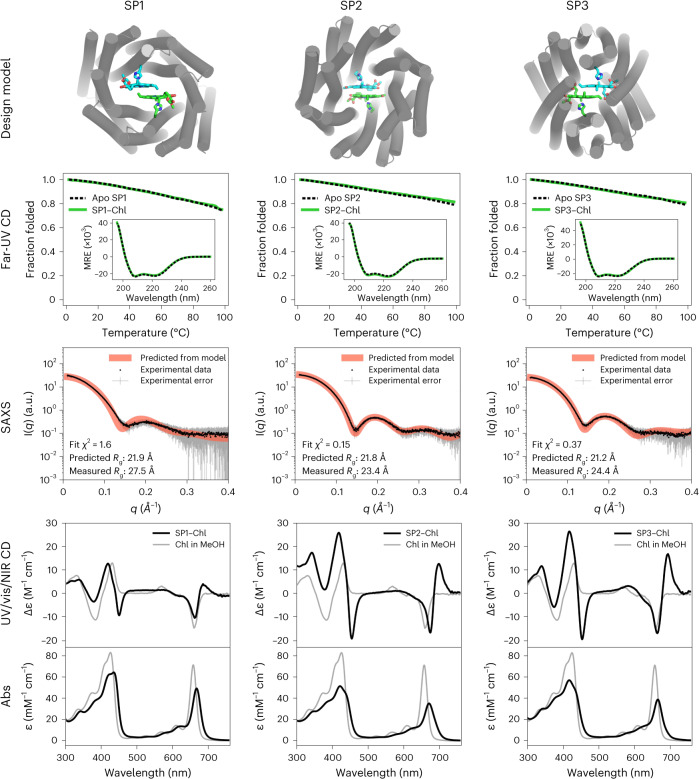
Folding, stability and ZnPPaM binding of special pair proteins. Top, special pair protein design models are displayed with α-helices represented as cylinders and Chl molecules as sticks. Second row, far-UV CD signals at 222 nm monitored with increasing temperature show that proteins are highly thermostable in both apo states (black dashed traces) and ZnPPaM-bound states (green traces). Far-UV CD spectra measured at 25 °C (inset) have features typical of highly α-helical proteins, including minima at 208 and 222 nm. Binding of ZnPPaM has little effect on the secondary structure composition (green traces). Molar residue ellipticity (MRE) on the *y* axis is given in deg cm^2^ dmol^−1^ per residue. Third row, experimental SAXS data, presented as mean scattered intensity (I(q), black dots) ± s.d. (gray error bars), are in good agreement with SAXS profiles predicted from apo-state design models using the FoXS server, shown as red traces^38,39^. Bottom, the UV/vis/NIR CD spectrum of each protein in the ZnPPaM-bound state is shown compared to the control spectrum of ZnPPaM in methanol (MeOH) with the absorbance (Abs) spectrum of the same sample below. In each case, the protein-bound dimer acquires a positive band at ~690 nm, which is consistent with calculations based on dimer geometries (vide infra). Unbound ZnPPaM was removed by sterile filtration and PD-10 column chromatography before data collection. a.u., arbitrary units. Source data

Following special pair protein expression in *Escherichia coli*, SDS−PAGE gels showed that all 48 designs were present in the soluble fractions of the lysates. Proteins were purified using Ni-NTA agarose and size-exclusion chromatography (SEC) (Supplementary Fig. 1). All SEC traces exhibited protein absorption at the elution volume expected for homodimer formation. Of the 20 designs investigated by small-angle X-ray scattering (SAXS) in the apo state, 15 had SAXS profiles that suggested a three-dimensional (3D) shape consistent with the design model (Fig. 2, Supplementary Fig. 2 and Supplementary Table 2)^38,39^. The slightly lower predicted radius of gyration (*R*_g_) value compared with the experimental SAXS data is likely due to a dense hydration shell around the highly charged special pair proteins^40,41^. The far-ultraviolet (UV) circular dichroism (CD) spectra of three special pair proteins that were expressed in high yield (≥140 mg l^−1^) showed that these proteins were highly α-helical with and without the synthetic Chl *a* derivative Zn-pheophorbide *a* methyl ester (ZnPPaM). Thermal denaturation curves monitored by the CD signal at 222 nm indicated that all three proteins are highly thermostable in the apo and holo states (Fig. 2).

At longer wavelengths in the UV/visible/near-infrared (UV/vis/NIR) range, CD spectra can serve as a convenient probe of excitonic interactions between Chl molecules. Monomeric Chl molecules, including Chl *a* and ZnPPaM, exhibit asymmetric negative CD signals in the Q_y_ region near ~670 nm (Supplementary Fig. 3)^42^. However, when Chl dimers are arranged in chiral protein environments, excitonic interactions can produce delocalized transitions with chiral character, yielding CD signals that are stronger and conservative (that is, composed of a bisignate doublet that integrates to zero). Figure 2 shows that ZnPPaM molecules bound to the special pairs SP1, SP2 and SP3 proteins had bisignate CD features in the *Q*_*y*_ region (the red part of the spectrum), consistent with excitonic coupling between the Chl molecules. As shown in Supplementary Table 3, the *Q*_*y*_ CD features of SP2 and SP3 were substantially stronger relative to their *Q*_*y*_ absorption bands than the *Q*_*y*_ CD signal of monomeric ZnPPaM in organic solvent. ZnPPaM binding titrations of SP2 and SP3 monitored by CD in the *Q*_*y*_ region showed that the CD doublets are attributable to the binding of ZnPPaM dimers. Curve fitting of the CD titrations yielded SP2−ZnPPaM dissociation constants (*K*_d_) of 300 nM for *K*_d1_ and 2.5 μM for *K*_d2_, and SP3−ZnPPaM *K*_d_ values of 800 nM for *K*_d1_ and 1.0 μM for *K*_d2_ (Supplementary Fig. 4). Because of the lower CD signal of the SP1−ZnPPaM complex, we instead used absorbance and fluorescence to measure the SP1−ZnPPaM interaction. Absorption titrations yielded curve fits with *K*_d1_ and *K*_d2_ values of 290 nM and 430 nM for SP1, 110 nM and 2.0 μM for SP2 and 350 nM and 940 nM for SP3, respectively (Supplementary Figs. 5 and 6). Fluorescence titrations analyzed using a 1:1 binding model of protein monomer to ZnPPaM yielded *K*_d_ estimates of 660 nM for SP1, 480 nM for SP2 and 120 nM for SP3; these *K*_d_ values approximate the average of *K*_d1_ and *K*_d2_ for each protein (Supplementary Fig. 7).

### High-resolution X-ray crystal structures

**Fig. 3:**
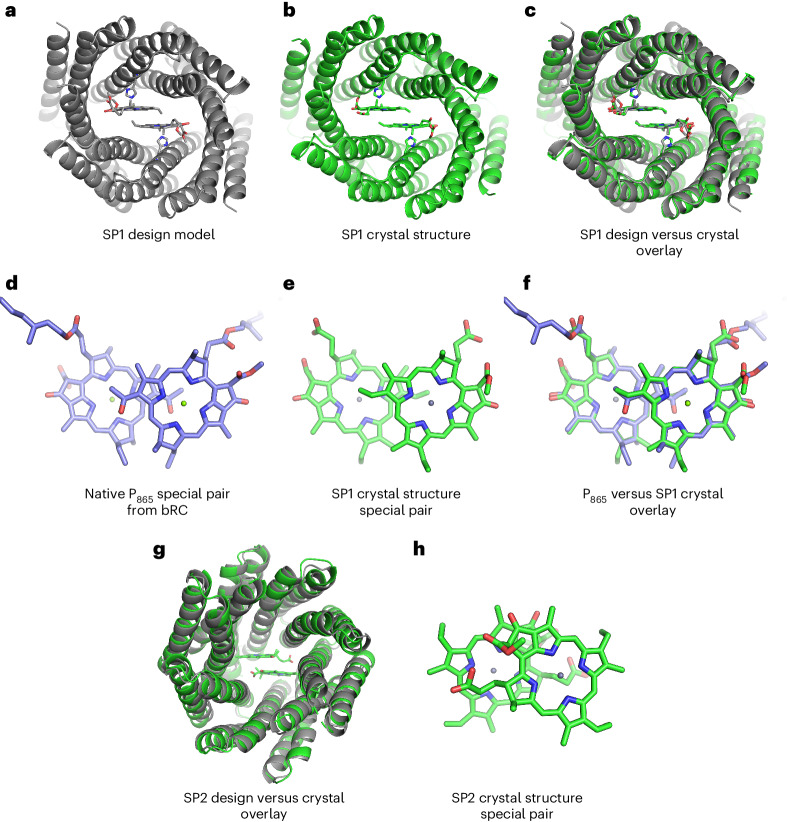
X-ray crystal structures of the designed special pair proteins. **a**, Rosetta design model of SP1. **b**, SP1 crystal structure at 2.0 Å resolution with ZnPPaM molecules bound (PDB 7UNJ). **c**, The SP1 Rosetta design model (gray) aligns to the SP1 crystal structure (green) with 1.6 Å Cα atom r.m.s.d. **d**, The BChl *a* special pair from a 2.5 Å-resolution cryo-EM structure of the purple bacterial LH1−RC complex (*R. sphaeroides*) (PDB 7PIL)^46^. **e**, The ZnPPaM dimer from the SP1 crystal structure shown in **b**. **f**, The ZnPPaM dimer of the SP1 crystal structure (green) aligns with the native purple bacterial special pair (blue) to 0.23 Å r.m.s.d. across corresponding atoms of the tetrapyrrole rings. **g**, The Rosetta design model of SP2 (gray) aligns to the holo-state SP2 crystal structure (green, PDB 7UNI) with 1.4 Å C_α_ atom r.m.s.d. **h**, The ZnPPaM dimer in the SP2 crystal structure (green) deviates from the predicted dimer geometry (not shown; 3.5 Å r.m.s.d.).

Based on the results of the SEC, SAXS and spectroscopic experiments (Fig. 2 and Supplementary Figs. 1–7), we selected promising candidates for X-ray crystallographic structure determination. We solved the crystal structures of SP1 and SP2 and found that both had protein backbone conformations that matched the corresponding design models to within 1.7 Å Cα root mean square deviation (r.m.s.d.) (Fig. 3).

The X-ray crystal structure of SP1 was solved in the ZnPPaM-bound state to 2.0 Å resolution, revealing a special pair geometry closely matching that of purple photosynthetic bacteria (Fig. 3a–f). The rotameric state of the Zn-ligating His121 is identical to that in the design model, and several hydrophobic and T-stacking interactions form as designed. Hydrogen bonds to the ring E ketone group, which has been shown to be important for modulating special pair redox potentials^43^, form with Gln10 in both ZnPPaM molecules, in agreement with the design model. Alignment of the tetrapyrrole rings of the SP1−ZnPPaM dimer with nine native BChl *a* special pairs from different species of purple bacteria gave r.m.s.d. values of 0.23–0.28 Å (refs. ^44–51^). (See details of r.m.s.d. measurements in Methods.) For comparison, the special pairs of two crystal structures of the same *Thermochromatium tepidum* LH1−RC complex deviate from one another by 0.22 Å r.m.s.d. across the tetrapyrrole rings (Protein Data Bank (PDB) 3WMM and 5Y5S)^45,51^. The r.m.s.d. between the ZnPPaM dimer in the SP1 crystal structure and its design model is 0.25 Å.

SP2 was intended to assemble a ZnPPaM dimer with a conformation substantially different from that of native special pairs to investigate the effect of dimer geometry. The SP2 crystal structure was solved in both the apo state and the ZnPPaM-bound state to 2.4 and 2.5 Å resolution, respectively. The apo- and holo-state amino acid backbones both agree with the SP2 design model to within 1.4 Å r.m.s.d. (Fig. 3g). The holo-state crystal structure has two copies of the SP2 dimer in the asymmetric unit; alignment of the two ZnPPaM dimers shows that their binding geometries are equivalent, with an r.m.s.d of 0.22 Å over the tetrapyrrole rings. The ZnPPaM molecules are ligated by His178, as in the SP2 design model. After alignment of the crystal structure and design model protein backbones, the corresponding tetrapyrrole rings are approximately coplanar. Despite the accuracy of the protein backbone design, the crystal structure shows that the ZnPPaM molecules are rotated and translated relative to the design model (3.5 Å r.m.s.d. across tetrapyrrole ring atoms). Compared with the apo-state crystal structure, the SP2 binding cavity widens by ~1.6 Å in the presence of ZnPPaM; this expansion provides the extra volume needed for the ZnPPaM molecules to adopt their unexpected conformation. While the ZnPPaM dimer in SP2 differs from the design model, the crystal structure nevertheless satisfies the objective of creating a non-native dimer geometry.

We did not succeed in solving the holo-state structure of SP3; however, we were able to solve a 3.05 Å-resolution apo-state structure of the closely related design SP3x, which shares 94% sequence identity with SP3. The SP3x homodimeric design model agrees with the X-ray crystal structure to 1.61 Å Cα r.m.s.d. (Supplementary Fig. 8).

### Excitonic coupling between Chl molecules

**Fig. 4:**
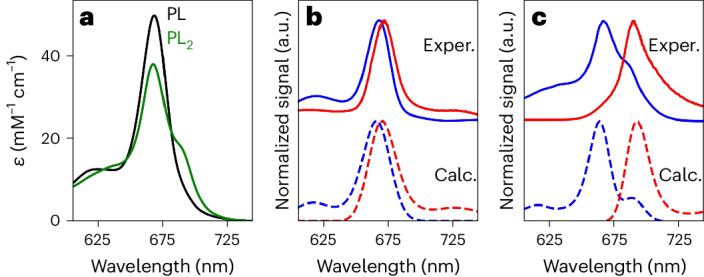
Spectral shift on ZnPPaM dimer binding in the SP2 protein. **a**, Molar absorptivity in solution of ZnPPaM when bound to SP2 as a monomer (PL; black trace) with 0.3 equivalents of ZnPPaM present per SP2 dimer and as a dimer (PL_2_; green trace). Monomer and dimer traces are fitted spectra from a binding titration experiment (Supplementary Fig. 5). **b**,**c**, Experimental (upper curves) and simulated (lower curves) absorption (blue) and fluorescence (red) spectra of PL (**b**) and PL_2_ (**c**) complexes at 75 K. Experimental data are for SP2 suspended in a sucrose/trehalose matrix with the same protein-to-ZnPPaM ratios as in **a**. Samples were photoexcited at 405 nm for fluorescence. Spectra in **b** and **c** are normalized to the unit peak intensity. Simulated spectra were generated in the PigmentHunter application^54^ with excitonic splitting in **c** induced by an interpigment coupling of 241 cm^−1^ as estimated by density functional theory. (See Methods and Supplementary Fig. 9 for details). Source data

The absorption and fluorescence spectra of native special pairs are shifted compared with those of monomeric BChl or Chl molecules, in part due to excitonic coupling between the BChl or Chl molecules, which enables them to act as exciton traps^5,6,52,53^. Absorbance, fluorescence and circular dichroism measurements along with time-dependent density functional theory calculations suggest that the chlorophyll molecules in our special pair proteins are also excitonically coupled. The SP2−ZnPPaM dimer absorbance spectrum exhibited splitting of the *Q*_*y*_ band in solution. Analysis of SP2−ZnPPaM absorbance in binding titrations (Fig. 4a and Supplementary Fig. 5) showed that the *Q*_*y*_ transition of monomeric ZnPPaM in SP2 had an absorbance maximum at 669 nm with an extinction coefficient (*ε*_669nm_) of 49,900 M^−1^ cm^−1^, whereas the SP2−ZnPPaM dimer spectrum had its *Q*_*y*_ maximum slightly shifted to 668 nm with a decreased *ε*_668nm_ of 38,200 M^−1^ cm^−1^. While the monomer had no discernable spectroscopic feature at 690 nm (its *ε*_690nm_ was 9,400 M^−1^ cm^−1^), the SP2−ZnPPaM dimer spectrum had a distinct shoulder with *ε*_690nm_ of 17,700 M^−1^ cm^−1^.

To investigate the origin of the SP2−ZnPPaM bands at 668 and 690 nm and rule out the possibility that they represent two populations of distinct ZnPPaM oligomers, we collected low-temperature absorption and fluorescence spectra (Fig. 4b,c and Supplementary Fig. 9; see Methods for details). We prepared a dimer sample with 2.0 molar equivalents of ZnPPaM per SP2 protein dimer and a monomer sample with only 0.3 molar equivalents per protein dimer, both in sucrose/trehalose films at 75 K. The monomer sample lacked a red-shifted shoulder (Fig. 4b), but in the SP2−ZnPPaM dimer sample, two emission bands were observed at 673 and 692 nm (Fig. 4c), consistent with excitonic coupling. Time-dependent density functional theory calculations were also consistent with excitonic coupling. Using the SP2−ZnPPaM dimer crystal structure (Fig. 3h), we calculated an excitonic coupling energy of 241 cm^−1^ (Methods). The experimental SP2−ZnPPaM absorption features at 668 and 690 nm correspond to a *Q*_*y*_ peak splitting of 477 cm^−1^ (a coupling of 239 cm^−1^), which is consistent with the calculated value. We also simulated absorption and fluorescence spectra in the PigmentHunter application^54^ (dashed lines in Fig. 4b,c and Methods). The simulated dimer spectra successfully reproduced the weak oscillator strength of the lower-energy state and the large shift between the absorption and fluorescence maxima. The close agreement between the calculated and experimental data supports the conclusion that the SP2−ZnPPaM dimer system exhibits excitonic delocalization.

Calculations on the SP1−ZnPPaM dimer crystal structure (Fig. 3e) yielded a lower excitonic coupling of 87 cm^−1^. Experimental low-temperature spectra of SP1−ZnPPaM in 40% glycerol showed only a modest broadening of the SP1−ZnPPaM dimer fluorescence emission band compared with the monomer, consistent with weaker excitonic coupling (Supplementary Fig. 10).

Further confirmation of excitonic coupling was obtained by comparing experimental and calculated CD spectra based on the ZnPPaM dimer geometries of the design models and crystal structures. We found that the signs of the CD Cotton effects predicted from the crystal structures of SP1 and SP2 were consistent with the experimental signs in the *Q*_*y*_ region. In the SP3 dimer, the calculated spectrum based on the design model agreed with the experimentally measured signs of the CD Cotton effects, suggesting that the P_700_-like ZnPPaM dimer in SP3 assembles as designed. (See Methods and Supplementary Figs. 11–14 for details of CD spectral calculations and simulations of *Q*_*x*_ and Soret bands.)

### Special pair proteins as energy transfer acceptors

**Fig. 5:**
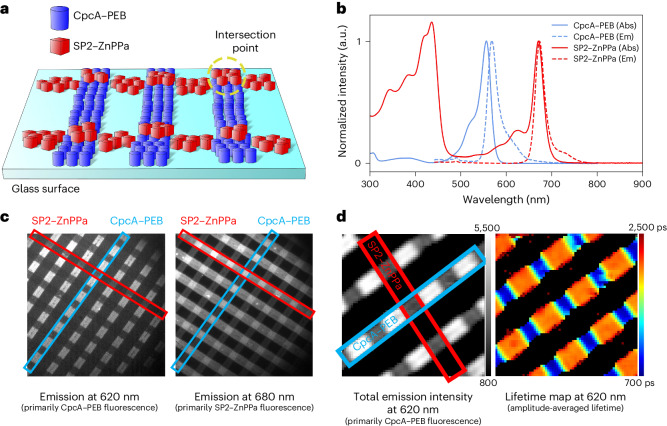
SP2 functions as an energy transfer acceptor for native light-harvesting proteins. **a**, Schematic of contact-printed proteins on a functionalized glass surface depicting lines of CpcA−PEB with separate rows of SP2−ZnPPa printed on top. **b**, Normalized absorbance (Abs) and fluorescence emission (Em) spectra of CpcA−PEB and SP2−ZnPPa. Spectra were collected in solution before contact printing on the glass surface. **c**, Fluorescence intensities of the printed cross-pattern of SP2−ZnPPa and CpcA−PEB at 620 nm and 680 nm with excitation by a 450-nm wide-field LED. **d**, Fluorescence intensity and lifetime map monitored at 620 nm with excitation by a 485-nm laser. Bars represent absolute fluorescence intensity in arbitrary units (a.u.) (left) and fluorescence lifetime in picoseconds (right). Source data

Native special pairs have critical roles as energy transfer acceptors for antenna proteins. To test whether our designed special pair proteins participate in energy transfer with natural light-harvesting proteins, we analyzed excitation energy transfer in two-dimensional (2D) surface arrays using fluorescence lifetime imaging microscopy (FLIM) along with nanoimprint lithography^55^ (Fig. 5). We selected the cyanobacterial antenna protein CpcA with attached phycoerythrobilin (PEB) as the energy transfer donor. CpcA−PEB was purified from *E. coli* and had a strong fluorescence emission maximum at 568 nm, with emission extending past 630 nm (ref. ^56^) and overlapping with the excitation spectrum of Zn-pheophorbide *a* (ZnPPa) when bound to SP2. *Q*_*y*_ peak splitting was not observed in SP2 assembled with ZnPPa instead of ZnPPaM (Fig. 5b), suggesting that the peripheral substituents of chlorin have a role in excitonic coupling. To monitor energy transfer, ~5-μm-wide linear arrays of CpcA−PEB and perpendicular linear arrays of SP2−ZnPPa were applied to a poly(l-lysine)-functionalized glass surface by contact printing^55^, creating intersection points where CpcA−PEB and SP2−ZnPPa interacted and other locations in which only one of the proteins was present. Wide-field epifluorescence imaging with excitation at 450 nm was used to analyze the surface attachment; filtering emission at 620 nm preferentially displayed regions of CpcA−PEB, whereas filtering at 680 nm preferentially showed SP2−ZnPPa. At the intersections between the lines, donor CpcA−PEB emission (620-nm filter) was decreased and SP2−ZnPPa acceptor (680-nm filter) emission was increased, indicating energy transfer from donor to acceptor (Fig. 5c).

To quantify the strength of the interaction between CpcA−PEB and SP2−ZnPPa, we used time-resolved single-photon counting. The surface was illuminated with a 485-nm picosecond laser filtered at 620 nm (donor emission) and photons were counted for individual pixels (surface resolution of approximately 300 nm) over time, allowing both total fluorescence intensity and lifetime to be analyzed (Fig. 5d). In regions with only CpcA−PEB, the fluorescence intensity was greater than 4,000 arbitrary units (a.u.) and the lifetime was more than 2 ns (*τ*_av_ = 2,058 ps). Regions with both CpcA−PEB and SP2−ZnPPa show reduced fluorescence intensity (< 1,000 a.u.) and lifetimes under 0.9 ns (*τ*_av_ = 839 ps). We estimated the energy transfer efficiency of CpcA−PEB to SP2−ZnPPa in 2D arrays to be 59%, similar to the efficiencies recorded for natural fluorescent proteins^57–59^ (see Supplementary Fig. 15 for time-resolved photon counting and energy transfer calculation).

### Chl-binding supercomplex

For efficient solar energy conversion, nature organizes photosynthetic machinery into specialized compartments such as thylakoids in plants or chromatophore vesicles in purple photosynthetic bacteria^60^. As a first step toward such structures, we sought to incorporate a Chl-binding special pair protein into a two-component supercomplex with octahedral symmetry^61^. The *C*_2_ symmetry axes of 12 copies of the SP2 dimer and the *C*_3_ axes of 8 copies of a *C*_3_-symmetric homotrimer^62^ were aligned with the *C*_2_ and *C*_3_ axes of an octahedron. We sampled rotations and translations along these axes to generate a closely packed octahedral model with the *C*_2_ dimers on the edges and the *C*_3_ trimers on the vertices. Interface residues were redesigned to create binding surfaces between the SP2 dimers and the trimers. Twenty-one designs were experimentally characterized, and one was found to assemble into octahedral structures by negative-stain EM (Supplementary Figs. 16 and 17). In this nanocage, the SP2-like component shares 87% sequence identity with the original SP2 design and its absorbance spectrum had a red-shifted shoulder in the *Q*_*y*_ region, similar to the original SP2−ZnPPaM complex (Supplementary Fig. 16).

**Fig. 6:**
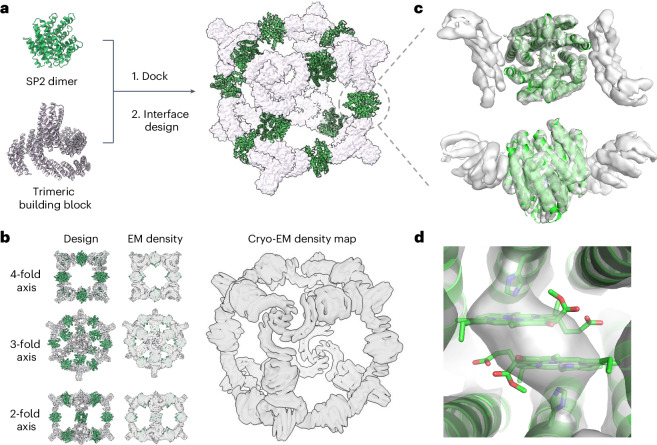
Cryo-EM structure of a nanocage that assembles Chl dimer proteins. **a**, The nanocage design model was generated by docking the SP2 dimer with a trimeric building block to form a two-component octahedral nanocage architecture. Rosetta sequence design was used to stabilize interfaces between the dimeric and trimeric building blocks. **b**, Multiple views of the nanocage design model (left), the design model docked into the cryo-EM density (middle) and cryo-EM density alone (right) (EMDB EMD-40208, map 1). The cryo-EM map (gray) closely matches the design model (color). **c**, Axial and lateral views of the *C*_2_ dimer unit. The holo-state crystal structure of SP2 (green, PDB 7UNI) was fitted into the EM density map of the nanocage (gray surface; EMDB EMD-40209, map 2). **d**, A close-up view of the Chl-binding pocket indicates that the ZnPPaM dimer structure in the nanocage EM density map (gray surface; map 2) is consistent with the SP2 crystal structure (green).

The cryo-EM structure of the 600-kDa octahedral nanocage with 24 ZnPPaM molecules bound to it was solved to 6.5 Å resolution, with the helices well resolved. The cryo-EM structure is very similar to the computational design model in both the protein−protein interfaces and the overall architecture (Fig. 6a,b and Supplementary Figs. 18 and 19). The asymmetric unit of the cryo-EM structure agrees with the design model to 3.4 Å backbone r.m.s.d. Variability analysis showed several modes of flexibility, which may have limited the resolution (see Supplementary Information for movies of protein breathing motions). Although the resolution is not sufficient to confidently determine the orientations of the Chl molecules, the cryo-EM density map is consistent with the Chl dimer geometry in the SP2 crystal structure (Fig. 6c,d).

## Discussion

We describe de novo-designed proteins that hold Chl dimers in precisely defined, closely juxtaposed geometries. We obtained crystal structures of two holo-state designs: the first, SP1, reproduces the binding geometry of the native purple bacterial reaction center special pair with sub-Ångstrom precision, and the second, SP2, has a distinct geometry with the Chl molecules closer together. Our use of symmetry reduces the complexity of the design procedure while ensuring equivalent site energies for the two bound Chl molecules to strengthen excitonic coupling. Symmetry also enables integration of the de novo special pair proteins into larger supercomplexes. Our octahedral nanocage incorporating 12 ZnPPaM dimers is a first step toward de novo design of photosynthetic compartments analogous to thylakoids or chromatophores.

SP2 exhibits spectroscopic hallmarks of native special pairs, including Cotton effects by CD, shifting of absorption and fluorescence bands and energy transfer activity when paired with the native antenna protein CpcA. SP1 exhibits weaker excitonic coupling than SP2 and the BChl special pair of purple photosynthetic bacteria, despite its close structural similarity to the latter. The stronger coupling of SP2 relative to SP1 may reflect the closer spacing of the ZnPPaM molecules in SP2, whereas the stronger coupling of the purple bacterial special pair is likely due to the stronger *Q*_*y*_ transition dipole moment of bacteriochlorins compared with chlorins^63^. Directed evolution could alter the binding specificity for different types of Chl molecules and increase absorbance band shifts for more effective exciton trapping. Prediction of the spectroscopic properties of a Chl dimer in a protein is complicated by the fact that Chl−Chl coupling energies are typically similar in magnitude to the available thermal energy, Franck−Condon active vibrational and phonon reorganization energies and local Chl vibrational frequencies^33^. Accurate optical predictions require benchmarking of theoretical methods using robust model systems. Native photosynthetic proteins can be difficult to isolate and typically contain many interacting pigment molecules, which creates spectral congestion. The highly thermostable, water-soluble Chl dimer proteins described here avoid the complexity of native pigment−protein complexes and provide a testbed to investigate structure−spectrum relationships.

Studies of photosynthetic light harvesting and charge separation indicate that natural photosynthesis leaves room for efficiency improvements^1,25,64–66^. The successful design of excitonically coupled chromophore pairs and their assembly into organized superstructures suggests that de novo protein design could provide a route to new solar-to-fuel energy conversion technologies. With its red-shifted absorbance spectrum, SP2 is well tuned to accept energy from light-harvesting Chl molecules or other pigments (Fig. 5), and it has the long-lived excited state (fluorescence emission lifetime of >3 ns; Supplementary Fig. 9) required to allow electron transfer to occur. To couple light absorption to charge separation for solar fuel production, the next step is to engineer the transfer of the excited-state electron to a low-potential electron acceptor.

## Methods

### Computational placement of the Chl special pair into symmetric protein scaffolds

Identifying residue positions capable of accommodating the Chl special pair, and that are scalable to millions of potential scaffolds, was achieved by using a motif-hash-based method^34,68^ specifically adapted for the His−Chl dimer motif inspired by the special pair of purple bacteria, P_865_. However, the number of example structures of the His−Chl dimer motif found in PDB is not acceptable for effectively populating a motif hash table. Therefore, additional structural examples of the symmetric His−Chl dimer complex were generated de novo.

The conformer generation was achieved using the NeRF algorithm (available on GitHub at https://github.com/atom-moyer/nerf), which translates internal molecular coordinates to global molecular coordinates. Various conformers were generated by varying the internal coordinates, such as the relative positioning of the Chl groups, the dihedral of ligation by the His residue, and the rotamer of the His side chain. The full complex was duplicated along the *C*_2_ axis to create the symmetric complex. If the relative orientations of the Chl molecules were varied, clashes between the rings and their substitutions were evaluated and filtered. The entire process of de novo motif generation was repeated for ligation with the epsilon and delta nitrogen atoms of the imidazole ring.

Once the de novo conformers were generated, the 6D transformation that defines the relative orientation of the N-Cɑ-C atoms of the ligating His residues was hashed using a method described previously^34,68^. The hashed 6D transformation was used as a key in a multivalue hash table (https://github.com/atom-moyer/getpy) and the associated value was a vector that defined the information necessary to rebuild the His−Chl complex, with nitrogen from the His residue used for ligation and the internal coordinates of the His rotamer.

During the evaluation of the design scaffolds, the 6D transformation of each symmetric residue pair across chains was evaluated and hashed using the same method used to hash the de novo conformers described above. This allowed the identification of symmetric residue pairs that had similar 6D transformations to the potentially acceptable ligation geometries. If a matching 6D transformation was found, the His−Chl complex was rebuilt from the associated value in the hash table and the complex was evaluated in the context of the protein. If the Chl molecules did not clash with the backbone atoms of the protein, the placement was accepted and passed into the protein design process.

A Python package and example scripts that generate the de novo hash tables and place the His−Chl complexes into symmetric proteins can be found at https://github.com/atommoyer/stapler.

### Protein expression and purification

Synthetic genes with N-terminal His_6_ tags followed by tobacco etch virus (TEV) protease cleavage sites were purchased in pET29b expression vectors from Integrated DNA Technologies. Plasmids were transformed into Lemo21(DE3) competent *E.* *coli* (New England Biolabs). For each protein, a single *E.* *coli* colony was grown in a culture of 5 ml LB with 100 μg ml^−1^ kanamycin overnight at 37 °C. Overnight cultures were used to inoculate 50- to 500-ml cultures of autoinduction medium^69^. Bacteria were grown in autoinduction medium at 37 °C with shaking for 4 h and then incubated with shaking overnight at 18 °C. Bacteria were harvested and resuspended in 300 mM NaCl, 30 mM imidazole, 25 mM Tris buffer at pH 8, ~0.01 mg ml^−1^ DNase (Sigma-Aldrich), ~0.1 mg ml^−1^ lysozyme (Sigma-Aldrich) and Pierce protease inhibitor tablets (Thermo Fisher Scientific). Bacteria were lysed by sonication and centrifuged at ~18,000*g* for 30 min. Soluble fractions were purified using immobilized metal affinity chromatography gravity columns packed with Ni-NTA agarose resin (Qiagen) at room temperature. Columns were washed with a buffer containing 20 mM imidazole and proteins were eluted with 300 mM imidazole buffer. Samples were digested with His-tagged TEV protease in the presence of 0.5 mM dithiothreitol for 1–2 days at room temperature. Digested proteins were buffer exchanged into 20 mM imidazole buffer, 300 mM NaCl and 25 mM Tris buffer at pH 8 and applied to immobilized metal affinity chromatography columns to remove TEV protease and uncleaved protein. Proteins were further purified by SEC using an ÄKTA FPLC instrument with a Superdex 200 Increase 10/300 GL column (GE Healthcare Life Sciences). Protein and Chl molecular weights were verified by reverse-phase liquid chromatography−mass spectrometry (LC−MS) using an Agilent G6230B time-of-flight instrument and AdvanceBio Desalting-RP column. Mass spectra were deconvoluted in BioConfirm using a total entropy algorithm (Supplementary Figs. 20–22).

### Protein−Chl sample preparation

ZnPPaM was purchased from Frontier Scientific. ZnPPaM stock solutions were prepared in dimethyl sulfoxide or methanol to concentrations between 200 μM and 1 mM. ZnPPaM concentrations were determined using mass measurements and the known absorptivity of Zn pheophytin *a*, which has a similar absorbance spectrum and an extinction coefficient *ε*_659nm_ of 77,300 M^−1^ cm^−1^ in 80% acetone/20% deionized water^70^. UV/vis absorbance spectra were acquired using a Jasco V-750 spectrophotometer with a bandwidth of 1 nm and scanning speed of 400 nm min^−1^. Protein−ZnPPaM complexes were prepared by slowly adding freshly prepared ZnPPaM stock solution to protein solution in aqueous buffer at room temperature and incubating the samples for several hours. Unbound ZnPPaM was removed by (1) centrifugation to pellet precipitated ZnPPaM and (2) sterile filtration using a 0.22-μm syringe filter and/or by PD-10 desalting column purification (Sephadex G-25 M resin, Cytiva Life Sciences).

### CD spectroscopy

CD spectra were collected using a Jasco J-1500 spectrophotometer. For protein secondary structure assays, spectra were measured on samples of 0.2–0.4 mg ml^−1^ protein in 1-mm quartz cuvettes from 260 to 190 nm with a 1 nm bandwidth, 1-nm data interval, data integration time (DIT) of 1 s and scanning speed of 50 nm min^−1^. Thermal melts were monitored at 222 nm from 2 to 98 °C with a 2-nm bandwidth and a DIT of 8 s. UV/vis/NIR CD transitions of protein-bound Chl molecules were examined in the 800- to 300-nm region in 1-cm quartz cuvettes as averages of 10 scans using a 3-nm bandwidth, 1-nm data interval, DIT of 4 s and scanning speed of 50 nm min^−1^, unless otherwise noted. UV/vis/NIR CD spectra shown in Fig. 2 were obtained after sterile filtering using a 0.22-μm filter and PD-10 desalting column purification (Sephadex G-25 M resin, Cytiva Life Sciences). Each spectrum represents the average of two independent sample preparations. Samples contained 8–15 μM protein (monomer concentration) with equimolar ZnPPaM, 150 mM NaCl and 10 mM Tris buffer at pH 8. ZnPPaM dry powder was dissolved in methanol stock solutions immediately before its addition to protein solutions. Samples were allowed to incubate for 8–16 h at room temperature before the spectra were measured.

### SAXS

Data were collected at the Advanced Light Source (ALS) at Lawrence Berkeley National Laboratory using the SIBYLS beamline for high-throughput SAXS^71^. Proteins were sent as 30-μl samples in 96-well plates with buffer-matched blank solutions for background subtraction. Datasets were processed in SAXS Frameslice v1.4.13 and compared with the design models using FoXS^38,39^.

### Fluorescence quantum yield measurements

Fluorescence spectra displayed in Supplementary Fig. 7 were recorded on a Fluorolog Horiba Jobin Yvon spectrofluorimeter equipped with a xenon lamp, a double monochromator and a photomultiplier detector. The experiments were carried out in a right-angle configuration. Each baseline-subtracted fluorescence spectrum was corrected for the spectral sensitivity of the fluorimeter and reabsorption by assuming that the middle of the cuvette is the origin of emission. Relative quantum yields were estimated using Chl *a* in diethyl ether as a ref. ^72^.

### Low-temperature absorbance and fluorescence spectroscopy in a sucrose/trehalose film

Solutions of SP2x−ZnPPaM were mixed with a saturated sugar solution made by dissolving 50:50 sucrose/trehalose (w/w) in distilled water as described previously^73^. A 100-μl sample of SP2 at 34 mg ml^−1^ (ZnPPaM dimer) or 156 mg ml^−1^ (ZnPPaM monomer) was added dropwise to 100 μl of the sugar solution and gently mixed. The sugar/protein mixture was dropped onto a 0.1-mm quartz cuvette (Starna Cells) and kept under vacuum in the dark for 24 h. The sample was then loaded into a Janis ST-100 cryostat using a custom-built copper cuvette holder and cooled with liquid nitrogen. A Lakeshore 330 autotuning temperature controller was used to control the temperature. An Agilent Cary-60 spectrometer was used to collect absorbance spectra at different temperatures. For the temperature-dependent fluorescence emission spectra, we used a home-built setup equipped with a Thorlabs 405-nm laser head (CPS405). The collected emission was fiber-coupled into a Flame Ocean Optics spectrometer. Lifetimes were recorded using a home-built all-reflective epifluorescence system. The samples were excited via a pulsed laser output from a 405-nm pulsed diode laser (LDH-P-C-405, PicoQuant) with a repetition rate of 10 MHz. The emission was subsequently filtered using a 420-nm long-pass dichroic beam splitter (DMLP425R, Thorlabs) and a 420-nm long-pass filter (10CGA-420, Newport). Emission was detected by avalanche photodiodes (PD050-CTD, Micro Photon Devices). Time-correlated single-photon counting traces were histogrammed using a Picoquant HydraHarp 400 and analyzed using the corresponding software.

### Molecular dynamics simulations

All molecular dynamics simulations were performed with Amber18 (ref. ^74^) using the ff14SB forcefield^75^ for proteins and TIP3P^76^ for water. To obtain the forcefield parameters of the chromophore (ZnPPaM), we used the MCPB.py module of Amber^77^. Atomic charges were calculated using the restrained electrostatic potential (ESP) fitting scheme, while force constants were calculated using the Seminario method. Quantum mechanical geometry optimization and ESP calculations were performed using Gaussian 16 (rev B.01)^78^ at the B3LYP/6–31 G* level. Parameters for the organic part of the chromophore were obtained from the general AMBER forcefield.

As starting structures, either design models or crystal structures were used. Protonation states were determined using the H++ webserver at pH 8 (using default parameters)^79–81^. Topology and geometry files were generated using LEaP with an isometric truncated-octahedron shape for the periodic box and a minimum distance of 1.5 nm between the protein and the edges of the box. Protein charges were neutralized with Na^+^ and Cl^−^ ions.

Minimization and initial equilibration steps were performed following a recently developed protocol^82^. Briefly, the protocol consists of nine sequential energy minimizations and short molecular dynamics runs, which sum to 4,000 steps of minimization and 40,000 molecular dynamics steps (totaling 30 ps), followed by a final molecular dynamics equilibration (500,000 steps, 1,000 ps). Then, after discarding the first 200 ns, production runs were done in the isothermal−isobaric (NPT) ensemble at 300.0 K with a time step of 2 fs, and bonds involving hydrogen atoms were constrained via the SHAKE algorithm. Constant temperature and pressure were ensured using the Langevin thermostat (collision frequency, 2 ps^−1^) and Monte Carlo barostat, respectively. Long-range electrostatics were considered via the particle mesh Ewald model, setting the direct space sum cutoff to 1.0 nm.

### Calculation of Chl dimer excitonic coupling and spectra

Calculations involving excited states were performed on the chromophore geometries of the design models and crystal structures. In the latter case, hydrogen atoms were added using UCSF Chimera 1.11 (ref. ^83^). Electronic couplings were calculated using the EET (electronic energy transfer) module from Gaussian, at the CAM-B3LYP/6-31 G* level. Environmental effects were considered through the polarizable continuum model^84,85^, choosing *n*-octanol as representative of the protein dielectric behavior. The EET analysis considered six singlet excited states per chromophore.

To obtain CD spectra, we used the results of the EET calculations and the Excitonic Analysis Tool program^86–88^. Rotatory strengths were calculated by considering both electric and magnetic dipoles in the velocity formulation. Spectral line shapes were simulated as Gaussians, with a full-width at half-maximum of 350 cm^−1^ for the *Q*_*y*_ and *Q*_*x*_ transitions and 1,150 cm^−1^ for transitions in the Soret region. The spectra were shifted by −0.25 eV to reproduce the experimental position of the *Q*_*y*_ band.

Simulated absorption and fluorescence spectra in Fig. 4 were calculated in the online PigmentHunter application, which uses the standard Frenkel exciton approach of constructing and diagonalizing an excitonic Hamiltonian to yield system transition dipoles and frequencies. Explicit expressions for absorption and fluorescence spectra are reported in ref. ^89^, with the exception that PigmentHunter uses a single preset (temperature-dependent) spectral line shape for each excitonic transition. Line shape parameters for Fig. 4 were calculated at 75 K using the experimentally determined vibrational and phonon densities for Chl *a* in the CP29 complex^90^. Simulations in Fig. 4 are ensemble averaged over 10^6^ realizations of pigment site energies chosen randomly from a Gaussian distribution with full-width at half-maximum of 500 cm^−1^; calculated spectra are convolved with a 10 cm^−1^ Gaussian to eliminate high-frequency noise due to finite statistical sampling. To match the experimental data, mean pigment site energies were set to 14,925 cm^−1^ for monomer (PL complex) and 14,750 cm^−1^ for dimer (PL_2_). Pigment transition dipoles were calculated from chains A and B of the experimental crystal structure using the transition ESP method and parameters for Chl *a*^91^. For consistency with the calculated CD spectra described above, the intersite coupling was rescaled by a factor of 1.212 to a value of 241 cm^−1^. (PigmentHunter’s automatically calculated intersite transition ESP coupling, which includes an empirical scaling factor to account for protein dielectric effects, is slightly smaller, at 199 cm^−1^.)

### FLIM

FLIM was conducted on a home-built laser scanning time-resolved fluorescence microscope, as described previously^55^. The microscope was equipped with a 485-nm picosecond diode laser (PicoQuant, PDL 828) and a 450-nm LED (Thorlabs, M470L2) (wide-field illumination) as excitation sources. The excitation light was focused by a ×100 objective (PlaneFluorite, NA = 1.4, oil immersion, Olympus). The emitted light was filtered using a 495-nm dichroic beam splitter (Semrock) and 565/25-nm, 630/20-nm and 680/45-nm bandpass filters (Semrock) to remove the background excitation light. The microscope was fitted with a spectrometer (150 lines/mm grating, Acton SP2558, Princeton Instruments) and an electron-multiplying charge-coupled device camera (ProEM 512, Princeton Instruments) for emission spectrum acquisition and wide-field imaging. A hybrid detector (HPM-100-50, Becker & Hickl) was used for single-photon counting. The modulation of the excitation laser was synchronized with a time-correlated single-photon counting module (SPC-150, Becker & Hickl) for the lifetime decay measurement. The repetition rate of the laser was set at 1 MHz. The excitation laser power was adjusted to produce a fluence of approximately 2 × 10^14^ photons per pulse per cm. The instrument response function of the setup was approximately 130 ps. Fluorescence lifetime images were recorded by scanning the excitation laser over the sample using a piezo scanner. FLIM data were analyzed using OriginPro (OriginLab Corporation) and FLIMfit (www.flimfit.org).

### X-ray crystallography of SP1 and SP2

Crystals of SP1 and SP2 were grown using protein purified as described above. Protein samples dispensed in 1-μl drops at purification concentrations were mixed with an equal volume of a crystallization solution and set in hanging drops (refer to Supplementary Table 4 for conditions). Vapor-phase equilibration of the resulting drops against a 1-ml reservoir of the same crystallization solution resulted in the growth of crystals. The crystals were flash-cooled in liquid nitrogen. Diffraction data were collected on a Pilatus area detector at the ALS synchrotron facility at beamline 5.0.2 for SP1−ZnPPaM and SP2−ZnPPaM protein assemblies. Diffraction data for SP2 were collected using a Rigaku HyPix-6000HE hybrid photon counting detector at the Fred Hutchinson Cancer Center. The resulting datasets (Supplementary Table 4) extend to 2.0 Å, 2.4 Å and 2.5 Å resolution for SP1−ZnPPaM, apo-state SP2 and SP2−ZnPPaM, respectively. The asymmetric units of the SP1−ZnPPaM and apo-state SP2 structures each contained one complete dimer (two copies of a protein subunit) and the SP2−ZnPPaM structure had two dimers in the asymmetric unit.

Data were processed using HKL2000 (ref. ^92^) or Aimless^93^. Subunits were placed by using the molecular replacement algorithm in PHENIX^94^. Local rebuilding of all constructs was performed using Coot^95^, followed by refinement in PHENIX^94^. For the ZnPPaM-bound structures, the protein was built and refined completely with water molecules (excluding water molecules from the binding site) and other chemicals before manually fitting ZnPPaM into the remaining density. ZnPPaM energies were calculated using eLBOW^96^. The final values for *R*_work_/*R*_free_ are notated in Supplementary Table 4.

### X-ray crystallography of SP3x

All crystallization experiments for the SP3x protein were conducted using the sitting drop vapor diffusion method. Crystallization trials were set up in 200-nl drops using the 96-well plate format at 20 °C. Crystallization plates were set up using a mosquito crystal from SPT Labtech and then imaged using UVEX microscopes and UVEX PS-600 from JAN Scientific. Diffraction-quality SP3x crystals formed in 2.4 M sodium malonate dibasic monohydrate pH 7.0.

Diffraction data were collected at the ALS at beamline 5.0.1. X-ray intensities and data reduction were evaluated and integrated using XDS^97^ and merged/scaled using Pointless/Aimless in the CCP4 suite^98^. Structure determination and refinement starting phases were obtained by molecular replacement using Phaser^99^ and the designed model for the structures. Following molecular replacement, the models were improved using phenix.autobuild^94^; efforts were made to reduce model bias by setting rebuild-in-place to false and using simulated annealing and prime-and-switch phasing. The structures were refined in Phenix^94^. Model building was performed using Coot^100^. The final model was evaluated using MolProbity^101^. Data collection and refinement statistics are recorded in Supplementary Table 4. Data deposition, atomic coordinates and structural factors reported for the SP3x protein in this paper have been deposited in the PDB, http://www.rcsb.org/, with accession code 8EVM.

### Protein structure alignment

Protein crystal structures were compared to Rosetta design models by aligning corresponding backbone Cα atoms and calculating r.m.s.d. using TM-align^102^. BChl or Chl special pair geometries were compared using the align function in the PyMOL Molecular Graphics System (v2.5.2; Schrödinger). To facilitate comparison of the geometries of special pairs composed of different BChl or Chl types, any unimportant conformational differences, such as the rotameric states of peripheral substituents, were omitted and differences in the Mg(II) versus Zn(II) positions were neglected, so that only the atoms of the tetrapyrrole rings were considered in pairwise special pair structural alignments. These atoms included the 4 pyrrole nitrogen atoms, 16 pyrrole carbon atoms and 4 methine bridge carbon atoms from each BChl or Chl monomer, giving 48 atoms per dimer that were used for structural comparisons. Corresponding atoms were aligned in PyMOL and the r.m.s.d. over all 48 atom pairs was calculated. Native BChl *a* special pairs used for comparison with the SP1 protein came from five different species of purple photosynthetic bacteria, including *Rhodobacter sphaeroides*, *Rhodopseudomonas palustris*, *Thermochromatium tepidum*, *Gemmatimonas phototrophica* and *Thiorhodovibrio* strain 970. The PDB IDs of the nine X-ray crystal and cryo-EM structures containing the native special pairs used for comparison with SP1 were as follows: 7PIL, 7VNY, 6Z27, 6Z02, 6Z5S, 3WMM, 5Y5S, 7O0U and 7C9R (refs. ^44–51^).

### Nanocage design

The Chl-binding dimer SP2 was docked against a library of trimeric cyclic oligomer scaffolds (*C*_3_) from previous de novo designs^34,62,103^ to form octahedral cages (*O*_32_) using RPXDock software^104^. The RPXDock package uses a hierarchical sampling strategy to search for interfaces with high shape complementarity based on residue pair transform scoring. The ten docking configurations with the best score for each scaffold were subsequently sequence designed by symmetric RosettaDesign calculations, using a previously reported protocol^61^ to carry out two-component protein−protein interface design. Briefly, we aimed to design low-energy, well-packed hydrophobic protein−protein interfaces where protein building blocks are treated as rigid backbones and only side chain rotamers of interface residues are packed with layer design restrictions. beta_nov16 or a clash-fixed score function was used during the design. Finally, all cage designs were filtered on the basis of shape complementarity (>0.6), interface surface area (solvent-accessible surface area, 1,000 < SASA < 1,600), predicted binding energy (dd*G* <−20 kcal mol^−1^), buried unsatisfied hydrogen bonds (uhb <3) and clash check (< 3). All Rosetta scripts used are available upon request.

### Transmission negative-stain EM and image processing

SEC-purified cage fractions were diluted to about 0.5 µM (monomeric component concentration) for negative-stain EM characterization. Briefly, on a glow-discharged formvar/carbon supported 400-mesh copper grid (Ted Pella), 6 μl of protein sample was drop-casted for 2 min. The grid was blotted and stained with 3 μl of 2% uranyl formate, blotted again and then stained with 3 μl of uranyl formate for 20 s before final blotting. Micrographs of stained samples were acquired using a 120-kV Talos L120C transmission electron microscope. All negative-stain EM datasets were collected using EPU software and processed using cryoSPARC^105^ with contrast transfer function (CTF) correction. All the particle picks were 2D classified for 20 iterations into 50 classes. Particles from selected classes were used to build the ab initio initial model. The initial model was homogeneously refined using *C*_1_ and the corresponding *O* symmetry.

### Cryo-EM grid preparation and data collection

Grids (QUANTIFOIL R 2/2 on Cu 300 mesh grids + 2 nm C) were vitrified using a Vitrobot Mark IV with a chamber maintained at 22 °C and 100% humidity. Grids were plunge-frozen into liquid ethane directly following application for 5 s of 3.5 μl of the ZnPPaM-loaded nanocage to the glow-discharged surface of the grid. Grids were screened at the New York University cryo-EM core facility using a Talos Arctica microscope operated at 200 kV equipped with a Gatan K3 camera. Data were then acquired using a Titan Krios microscope operated at 300 kV equipped with a Gatan K3 camera with a BioQuantum imaging filter (‘Krios 2’; New York Structural Biology Center). Data were acquired from duplicate grids using Leginon^106^ and preprocessed (2⨯ binned and motion-corrected with MotionCor2 (ref. ^107^)) within Appion^108^. Full data collection parameters are shown in Supplementary Table 5.

### Cryo-EM data processing and model building

Aligned and dose-weighted micrographs were imported to cryoSPARC v.3 (ref. ^105^) and processed using the workflow shown in Supplementary Fig. 18. During data collection, we noted a high proportion of damaged (compressed or fragmented) nanocage particles in areas of ice with a reported thickness of less than 40 nm. Curation of micrographs to exclude those with the thinnest ice and with CTF fit resolution lower than ~6 Å facilitated selection of intact nanocage particles. 2D classification was performed on manually selected particles to generate templates representing diverse views of the nanocage, but subsequent template-based picking tended to exclude rare particle views. Recovery of these rare views was improved by using a single template for picking representing the view most often missed in prior template-based picking efforts (Supplementary Table 6). Compared with picking using multiple templates, using a single, rare-view template improved the recovery of diverse particle views, which were then used as a training set for Topaz^109^. Picking with Topaz yielded diverse, well-centered nanocage particles. Data from each of the two imaged grids were picked separately using Topaz and the curated particles were then combined and further curated in 2D. This larger set of curated particles was used to retrain Topaz (204,039 versus 19,355 in the initial Topaz training set) on the full set of micrographs from both grids. Particles picked using this Topaz model were then curated by 2D classification, micrograph curation by ice thickness and CTF fit values and removal of duplicates.

A 200-micrograph subset from a single grid was used to generate an ab initio 3D reconstruction. Following iterative rounds of homogeneous and heterogeneous refinement, this map served as the initial 3D model for processing the full particle set from both grids. 3D refinement and classification yielded a map of the full nanocage with an average reported resolution of ~6.5 Å (as calculated in cryoSPARC using a gold-standard FSC cutoff of 0.143). O symmetry was imposed during the final round of refinement. Continuous conformational heterogeneity likely limited the resolution of the full nanocage map because discrete states were not readily separable by further 3D classification. Multiple modes of flexibility were visualized using cryoSPARC’s 3D variability analysis^110^, supporting the notion that the nanocage particles used in refinement were subject to compression/deformation (see Supplementary Information for movies of protein breathing motions). We then used partial signal subtraction and focused refinement to improve the resolution in the ligand-binding region of the cage (region enclosed in yellow mask, Supplementary Fig. 18 inset). Before partial signal subtraction, particles were expanded with *T* symmetry (the highest-order symmetry containing a complete Chl-binding dimer). The symmetry-expanded, partially subtracted particle set was then refined in *C*_1_ using local refinement in cryoSPARC.

The cryo-EM map of the full nanocage was used for real-space refinement of a model in Phenix^111^. Due to the intermediate map resolution, all residues were modeled as alanine and restraints (secondary structure, Ramachandran and noncrystallographic symmetry constraints) were imposed during refinement. The designed nanocage model was used as a starting point for refinement and individual chains were docked into cryo-EM maps using Chimera^83^ before hydrogen removal and truncation to polyalanine using phenix.pdbtools. Stubbed, docked models were then subjected to restrained real-space refinement in Phenix. We observed a notable difference between the design model and the cryo-EM density in the angle between each trimeric interface helix and its attached DHR ‘arm’. To generate a starting model for restrained refinement of the full nanocage, we first performed rigid-body refinement, with each trimer subunit modeled as two rigid bodies (corresponding to the interface helix and ‘arm’ regions; residues 259–337 and 1–258, respectively). Cryo-EM model statistics are listed in Supplementary Table 7.

### Reporting summary

Further information on research design is available in the Nature Portfolio Reporting Summary linked to this article.

## Online content

Any methods, additional references, Nature Portfolio reporting summaries, source data, extended data, supplementary information, acknowledgements, peer review information; details of author contributions and competing interests; and statements of data and code availability are available at 10.1038/s41589-024-01626-0.

## Supplementary information

Supplementary Information Supplementary Figs, 1–22 and Tables 1–7.

Reporting Summary

Supplementary Video 1 Movie showing cryo-EM data of nanocage breathing motions #1.

Supplementary Video 2 Movie showing cryo-EM data of nanocage breathing motions #2.

## Source data

Source Data Fig. 2 Raw SAXS data for SP1, SP2 and SP3 apo proteins.

Source Data Fig. 2 CD and UV/vis spectra and thermal melts monitored by CD of SP1, SP2 and SP3 apo- and holo-state proteins.

Source Data Fig. 4 Low-temperature absorbance and fluorescence spectra of SP2−ZnPPaM.

Source Data Fig. 5 Absorbance and fluorescence spectra of CpcA−PEB and SP2−ZnPPa.

## Data Availability

X-ray crystallographic coordinates and data files of designed special pair dimer proteins were deposited at the PDB with accession codes 7UNJ (SP1 with ZnPPaM bound), 7UNH (SP2, apo state), 7UNI (SP2 with ZnPPaM bound) and 8EVM (SP3x, apo-state). All previously published high-resolution structures referenced in this manuscript, including the *Blastochloris viridis* LH1−RC complex (PDB 6ET5), are available at the PDB. An electron microscopy map of the full ZnPPaM-binding nanocage (map 1) was deposited in the EMDB with accession code EMD-40208 and a backbone model was deposited in the PDB with accession code 8GLT. An electron microscopy map of the ZnPPaM-binding region of the nanocage (map 2) was deposited in the EMDB with the accession code EMD-40209. Computational data related to molecular dynamics simulations and CD calculations have been deposited in the ioChem-BD database^112^ and are accessible at 10.19061/iochem-bd-6-268. Source data are provided with this paper.

## Code availability

The Rosetta macromolecular modeling suite (https://www.rosettacommons.org) is freely available to academic and noncommercial users. Commercial licenses for the suite are available from the University of Washington Technology Transfer Office. A Python package and example scripts for Chl docking can be found at: https://github.com/atommoyer/stapler. RPXDock scripts and computational methods are available on GitHub at https://github.com/willsheffler/rpxdock.
