# Supplementary material for: De novo design of proteins housing excitonically coupled chlorophyll special pairs
Source: Nat Chem Biol. 2024 Jun 3;20(7):906–15. doi: 10.1038/s41589-024-01626-0 (PMC11213709; doi:10.1038/s41589-024-01626-0)
Supplement: Supplementary file 1 — Supplementary Figs, 1–22 and Tables 1–7. [file 41589_2024_1626_MOESM1_ESM.pdf]

nature chemical biology

Supplementary information https://doi.org/10.1038/s41589-024-01626-0

De novo design of proteins housing excitonically coupled chlorophyll special pairs

In the format provided by the authors and unedited

Supplementary Information Table of Contents

Supplementary Figure 1 | Size exclusion chromatography (SEC) traces of purified SP apo-proteins........................ 2
Supplementary Figure 2 | Small angle X-ray scattering (SAXS) data and predictions...3
Supplementary Figure 3 | CD and absorbance spectra of Zn pheophorbide a methyl ester (ZnPPaM) in organic solvents...4
Supplementary Figure 4 | SP2- and SP3-ZnPPaM binding titrations monitored by CD.. 5
Supplementary Figure 5 | SP2-ZnPPaM binding titration monitored by UV/vis absorbance....................................6
Supplementary Figure 6 | SP1- and SP3-ZnPPaM binding titrations monitored by UV/vis absorbance................... 7
Supplementary Figure 7 | ZnPPaM fluorescence titrations with SP1, SP2, and SP3 proteins...................................8
Supplementary Figure 8 | SP3x crystal structure and design model comparison..9
Supplementary Figure 9 | Temperature dependence of SP2-ZnPPaM absorbance and fluorescence in a sucrose/trehalose film...10
Supplementary Figure 10 | Experimental 77 K fluorescence spectra in 40% glycerol... 11
Supplementary Figure 11 | Calculated circular dichroism spectra, using either crystal structure or design geometries, for the SP1, SP2, and SP3 proteins with ZnPPaM dimer bound... 13
Supplementary Figure 12 | Calculated circular dichroism spectra for SP1-ZnPPaM using the crystal structure geometry (PDB ID: 7UNJ), and either full excitonic couplings, or scaled down to 20%... 14
Supplementary Figure 13 | Structural parameters from molecular dynamics simulations on the SP1 (left) and SP2 (right) proteins, across 1.25 μs runs... 14
Supplementary Figure 14 | Calculated absorbance spectra for SP2-ZnPPaM using the crystal structure geometry. 15
Supplementary Figure 15 | Energy transfer activity of designed special pair protein SP2 on a glass surface...........16
Supplementary Figure 16 | Design and characterization of chlorophyll-binding O32-15 nanocage......................... 17
Supplementary Figure 17 | Negative-stain electron microscopy of purified O32-15 nanocage............................... 18
Supplementary Figure 18 | Cryo-EM data processing workflow for ZnPPaM-loaded nanocage.............................19
Supplementary Figure 19 | Cryo-EM of ZnPPaM-bound nanocage... 20
Supplementary Figure 20 | Reverse-phase liquid chromatography/mass spectrometry of SP1-ZnPPaM verifies molecular weights of protein and pigment..21
Supplementary Figure 21 | Reverse-phase liquid chromatography/mass spectrometry of SP2-ZnPPaM verifies molecular weights of protein and pigment..22
Supplementary Figure 22 | Reverse-phase liquid chromatography/mass spectrometry of SP3-ZnPPaM verifies molecular weights of protein and pigment..23
Supplementary Table 1 | Amino acid sequences of designed chlorophyll special pair proteins............................... 24
Supplementary Table 2 | Small angle X-ray scattering (SAXS) analysis of radii of gyration (RG).......................... 27
Supplementary Table 3 | ZnPPaM CD signal intensities in SP proteins compared to organic solvent controls........ 28
Supplementary Table 4 | Summary of X-ray crystallographic data collection and refinement statistics.................. 29
Supplementary Table 5 | CryoEM data acquisition.. 30
Supplementary Table 6 | CryoEM data processing...31
Supplementary Table 7 | Statistics for nanocage polyalanine model fitted and refined to cryo-EM map (EMD-40208).. 32
Supplemental References..33

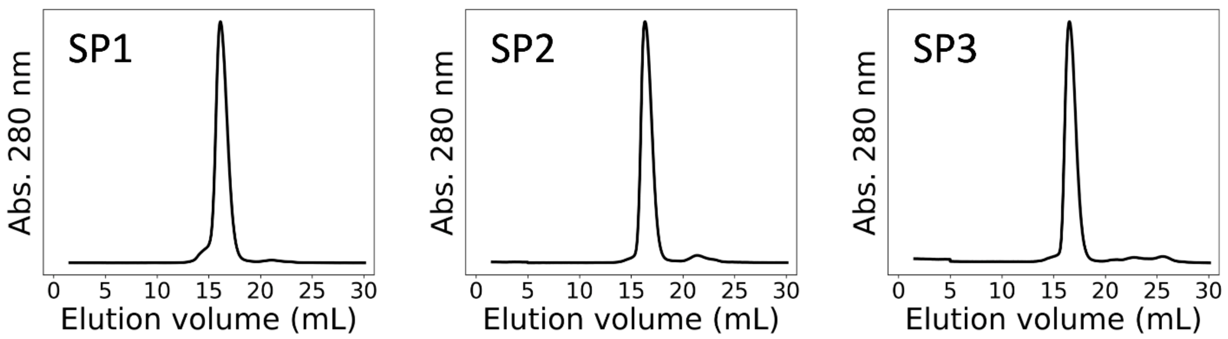

Supplementary Figure 1 | Size exclusion chromatography (SEC) traces of purified SP apo-proteins. Proteins were injected as 0.5 mL aliquots onto a Superdex™ 200 Increase 10/300 GL column (Cytiva Life Sciences). The narrow, isolated peaks indicate that the samples contain monodisperse protein. Running buffer was 150 mM NaCl, 10 mM Tris at pH 8. Proteins were purified by Ni-NTA and one round of SEC prior to collecting the data shown.

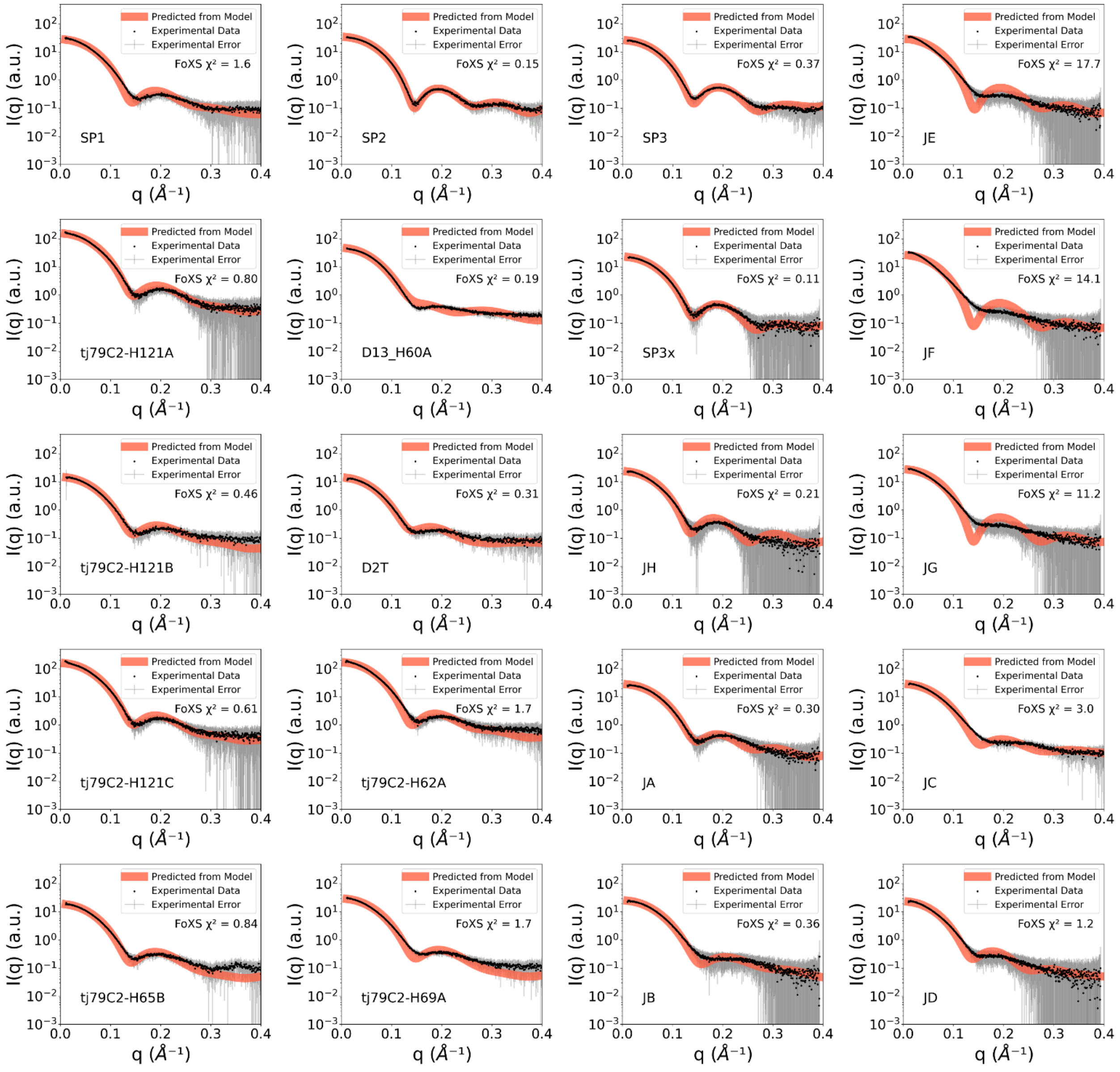

Supplementary Figure 2 | Small angle X-ray scattering (SAXS) data and predictions.
Data were collected on SEC-purified samples of 20-100 µM protein (dimer concentration) in the apo-state with 150 mM NaCl and 10 mM Tris at pH 8. Data were collected using the SIBYLS high throughput SAXS pipeline (Dyer et al. 2014). SAXS data are presented as mean values (black points) ± standard deviation (gray error bars), and SAXS profiles were calculated from apo-states of design models (red lines) using the FoXS server (Schneidman-Duhovny et al. 2013, 2016). Data sets with good agreement between data and FoXS prediction (as measured by χ^2 and radii of gyration, see Supplementary Table 2) are shown in the left 3 columns. These proteins likely fold as designed; SP1, SP2, and SP3x structures were verified by X-ray crystallography. The right column shows data sets with poorer agreement between SAXS data and predictions.

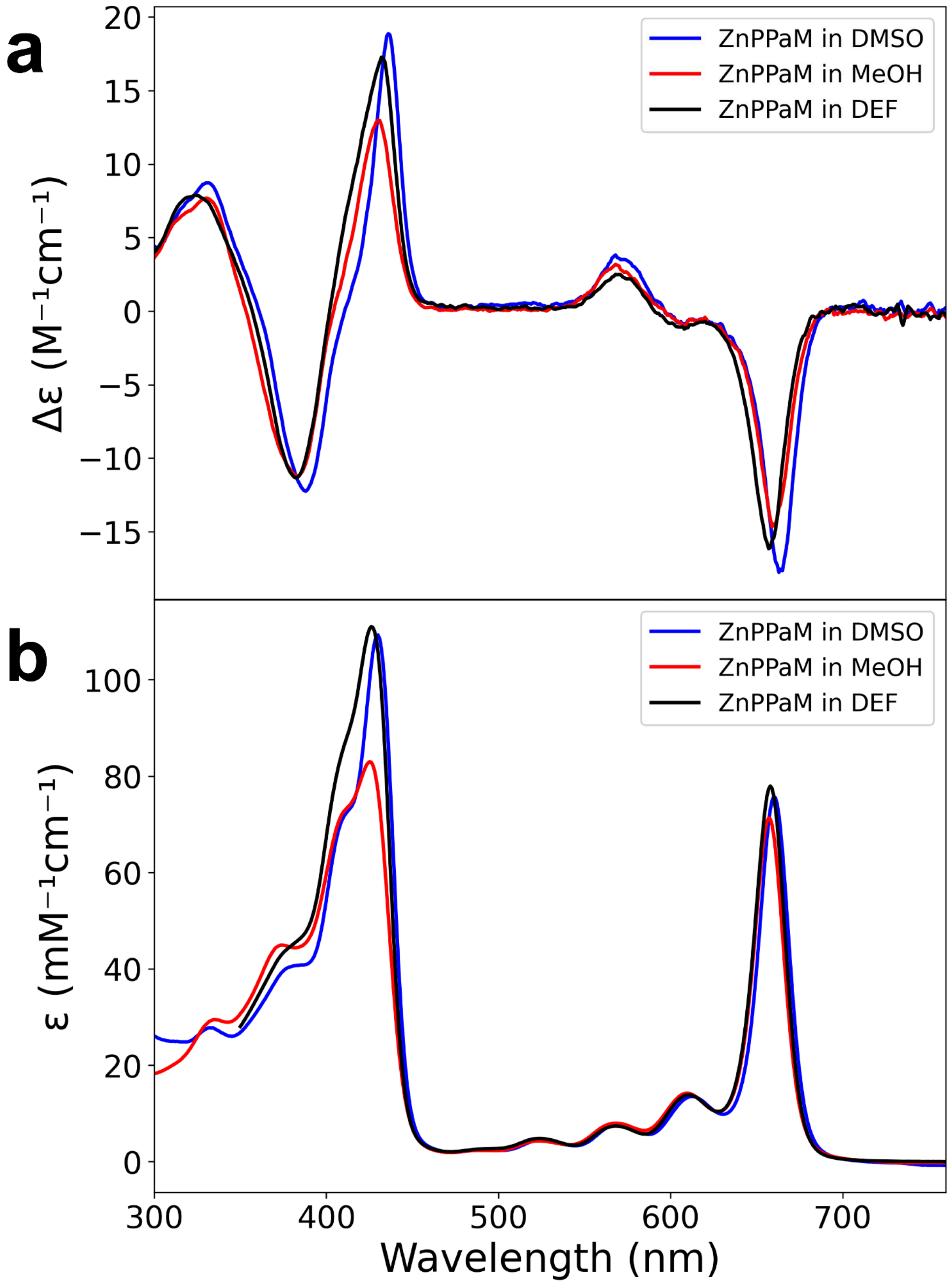

Supplementary Figure 3 | CD and absorbance spectra of Zn pheophorbide *a* methyl ester (ZnPPaM) in organic solvents.

(a) CD spectra of ZnPPaM (plotted as difference molar extinction coefficients, Δε) in dimethyl sulfoxide (DMSO), methanol (MeOH), and diethylformamide (DEF). Spectra were collected with 15 µM ZnPPaM in 1 cm cuvettes. Spectra of 1, 5, and 75 µM ZnPPaM in DEF (not shown) did not differ appreciably from the 15 µM ZnPPaM spectrum in DEF (black trace), meaning that there is no sign of concentration-dependent aggregation. All CD spectra were collected on a Jasco J-1500 CD spectrophotometer at 25°C. Each spectrum is the average of 10 scans using a 3 nm bandwidth, 50 nm/min scanning speed, 4 second data integration time, and a data interval of 1 nm. (b) Molar absorptivity (ε) of the same samples collected using a Jasco V-750 spectrophotometer with a 1 nm bandwidth, 400 nm/min scanning speed, 0.24 second ultraviolet/visible (UV/vis) response, and a data interval of 1 nm.

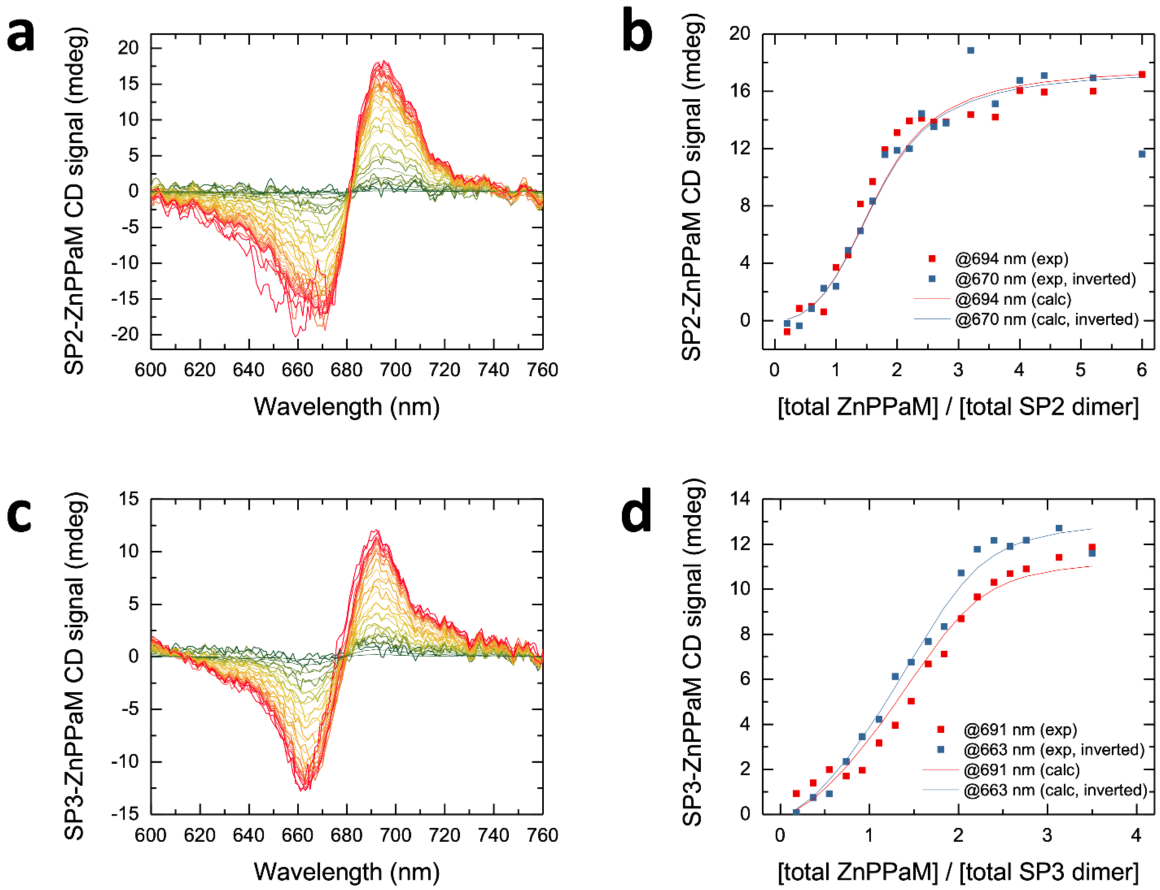

Supplementary Figure 4 | SP2- and SP3-ZnPPaM binding titrations monitored by CD.
(a) Measured and fitted CD spectra of 10.0 µM SP2 (dimer concentration) with increasing concentration of ZnPPaM. Titration was carried out in a 1 cm pathlength cuvette with 2.5 mL of 150 mM NaCl and 10 mM Tris at pH 8, and ZnPPaM was added from a DMSO stock solution. Approximately 20 minutes of incubation with stirring were allowed after each addition of ZnPPaM before the measurement of each spectrum. Spectra were measured on a Jasco J-1500 CD spectrophotometer at 25°C. Each spectrum was collected with a 1 nm bandwidth, 50 nm/min scanning speed, 2 second data integration time, and a data pitch of 1 nm. (b) Plots of the CD signal in panel (a) at 694 nm and the inverted CD signal at 670 nm (exp.) along with curve fits of the data (calc.). Curve fitting was performed under the assumption of a P + L ⟶ PL and PL + L ⟶ PL_2 mechanism in which the CD signal arises from PL_2. (P represents SP2 dimer protein and L represents ZnPPaM ligand). Fitting yielded SP2-ZnPPaM dissociation constant estimates of 320 nM for K_{D1} and 2.5 µM for K_{D2}. Fittings were performed with an algorithm that combines singular value decomposition and least squares regression, as implemented in ReactLab Equilibria. Estimates of K_{D1} values using this method are of low confidence due to the weak CD signal of PL. (c) SP3-ZnPPaM titration performed as in panel (a) with 10.9 µM SP3 (protein dimer concentration). (d) Plots and curve fitting of the SP3-ZnPPaM titration as in panel (b). Estimated SP3-ZnPPaM dissociation constants were 800 nM for K_{D1} and 1.0 µM for K_{D2}.

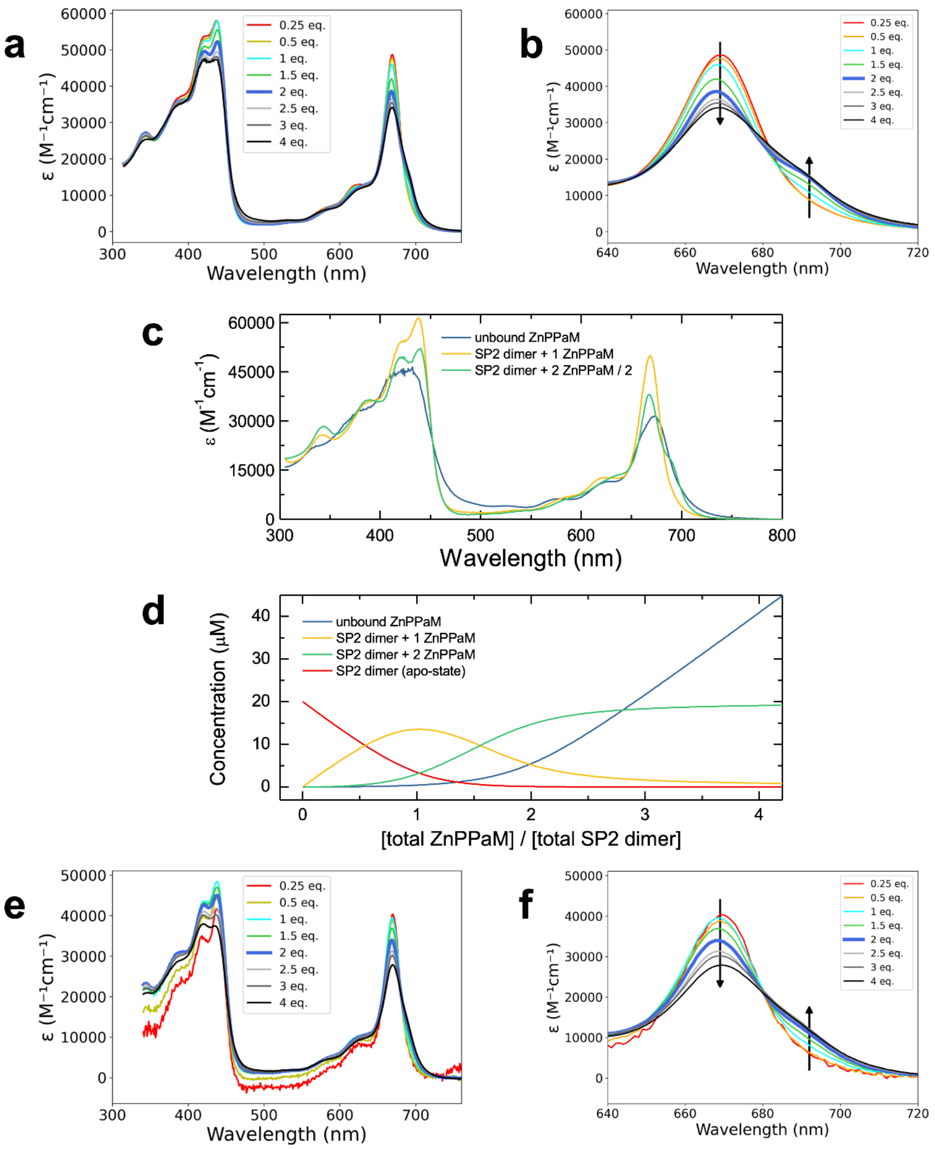

Supplementary Figure 5 | SP2-ZnPPaM binding titration monitored by UV/vis absorbance.
(a) UV/vis titration of ZnPPaM into a solution of 20 μM SP2 dimer (40 μM of SP2 monomer) in 150 mM NaCl, and 10 mM Tris at pH 8. Samples were prepared separately with 0.25 to 4 equivalents (eq.) of ZnPPaM per SP2 protein dimer (2.0 eq. ZnPPaM would be a stoichiometric amount). The y-axis is the molar extinction coefficient, ε, or OD divided by ZnPPaM concentration. ZnPPaM was added to protein samples from a DMSO stock solution. Samples were incubated overnight for 20 hours at 4°C before measurement at room temperature. (b) The same data as in panel (a) zoomed in on the Q_y region. Arrows indicate the direction ε changes as a function of increasing ZnPPaM concentration. (c) Fitted spectra and (d) species concentration vs. molar ratio of ligand to protein from fitting of the absorbance spectra in panels (a) and (b). During fitting, only the P + L → PL and PL + L → PL_2 reactions were considered, and ZnPPaM aggregation was disregarded. (P represents SP2 dimer protein and L represents ZnPPaM ligand). Curve fitting gives dissociation constants (K_Ds) of 110 nM and 2.0 μM for K_{D1} and K_{D2}, respectively. Fittings were performed with an algorithm that combines singular value decomposition and least squares regression, as implemented in ReactLab Equilibria. (e) Full spectra and (f) the Q_y region of a second titration with SP2 dimer protein concentration held at 2.0 μM. At 2.0 μM protein, the intensities of the spectral features at 669 and 692 nm depend upon the molar ratio of protein to ZnPPaM in a manner similar to the 20 μM protein titration shown in panels (a) and (b).

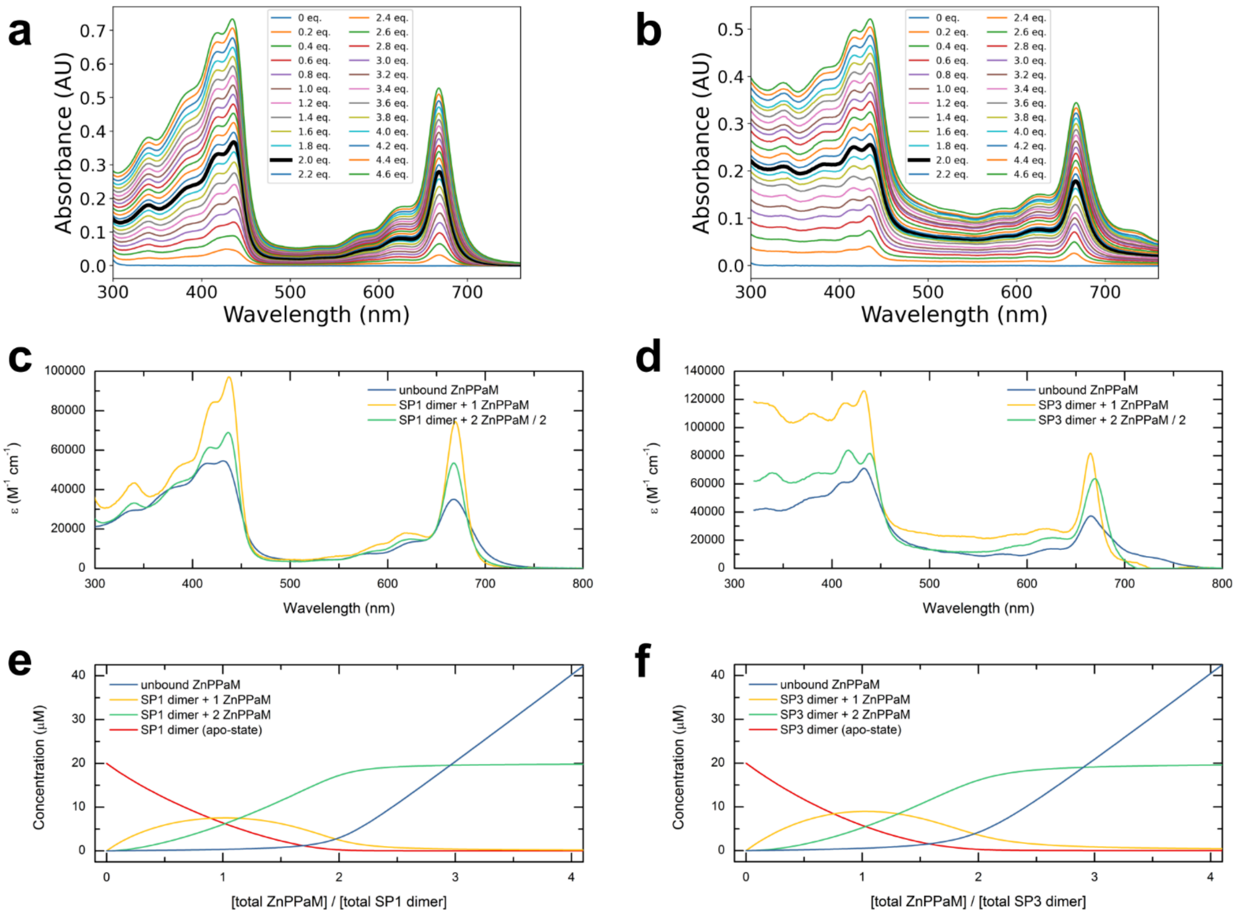

Supplementary Figure 6 | SP1- and SP3-ZnPPaM binding titrations monitored by UV/vis absorbance. UV/vis titrations of ZnPPaM were conducted using a solution of (a) 2.6 µM SP1 dimer (5.2 µM SP1 monomer) and (b) 1.4 µM SP3 dimer (2.8 µM SP3 monomer). ZnPPaM was added in increments of 0.2 eq. from a 1.03 mM DMSO stock solution (where 1 eq. is equal to the concentration of protein dimer). After each ZnPPaM addition, 10 minutes of equilibration with stirring were allowed before collecting an absorbance spectrum. Note that the SP3 titration had some evidence of scattering, presumably due to protein aggregation. Samples contained 150 mM NaCl and 10 mM Tris buffer at pH 8.0. Fitted absorption spectra for (c) SP1 and (d) SP3 and fitted species concentrations plotted against molar ratio of ligand to protein for (e) SP1 and (f) SP3 were obtained. Curve fitting gave an SP1-ZnPPaM K_{D1} of 290 nM, SP1-ZnPPaM K_{D2} of 430 nM, SP3-ZnPPaM K_{D1} of 350 nM, and SP3-ZnPPaM K_{D2} of 940 nM. Fits were obtained using singular value decomposition and least squares regression in ReactLab Equilibria. During fitting, aggregation was disregarded and only the P + L → PL and PL + L → PL_2 reactions were considered (where P is protein and L is ZnPPaM ligand).

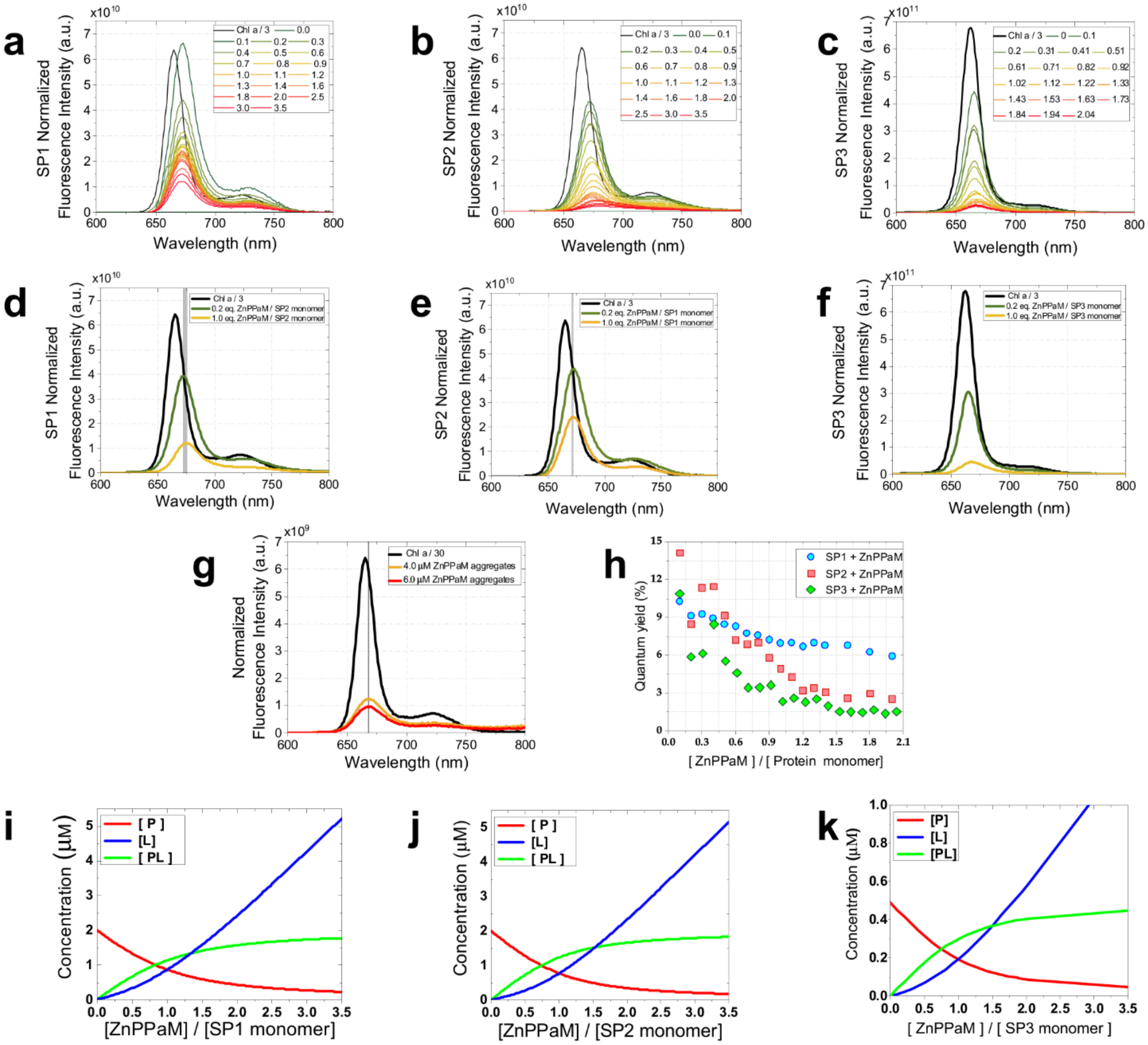

Supplementary Figure 7 | ZnPPaM fluorescence titrations with SP1, SP2, and SP3 proteins.
All spectra shown in panels (a) to (g) are normalized to pigment concentration. (a-c) Fluorescence emission spectra of 2.0 μM SP1, 2.0 μM SP2, and 0.49 μM SP3, respectively, with increasing molar equivalents (eq.) of ZnPPaM per protein monomer as indicated in the legends. Chlorophyll *a* (Chl *a*) fluorescence emission spectrum in diethyl ether (with its emission intensity divided by 3) is shown for comparison. The quantum yield of Chl *a* in ether is 32% (Weber and Teale 1957). Spectra were measured in 150 mM NaCl with 10 mM Tris buffer at pH 8. The excitation wavelength used was 644 nm, and spectral shape was reconstructed with excitation at 420 nm. (d-f) Fluorescence emission spectra of SP1, SP2, and SP3, respectively, with only 0.2 eq. and 1.0 eq. ZnPPaM spectra shown. (g) Fluorescence emission spectra of ZnPPaM without protein present shows fluorescence intensity is significantly lower for unbound ZnPPaM than protein-bound ZnPPaM. (h) Quantum yield of ZnPPaM fluorescence emission is plotted against ZnPPaM eq. per SP1, SP2, and SP3 protein monomer. (i-k) Concentration profiles of SP1-, SP2-, and SP3-ZnPPaM fluorescence titrations, respectively. The concentration profiles were constructed by fitting the quantum yield vs. [ZnPPaM]/[protein monomer] plots in panel (h) to the binding model P + L → PL, where P is the protein monomer and L is the ZnPPaM ligand. The curve fits gave K_D estimates of 660 nM for SP1-ZnPPaM, 480 nM for SP2-ZnPPaM, and 120 nM for SP3-ZnPPaM. These K_Ds represent approximations of the average K_D for the first and second ZnPPaM for each protein.

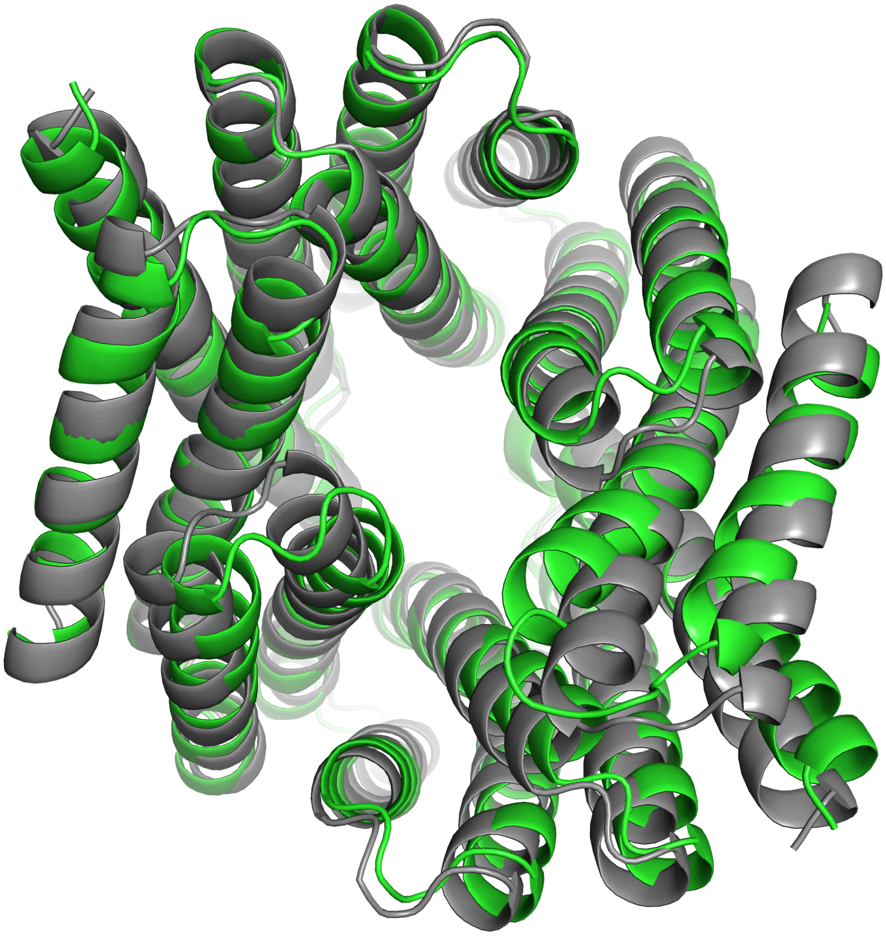

Supplementary Figure 8 | SP3x crystal structure and design model comparison.
The SP3x design model (gray) and apo-state X-ray crystal structure at 3.05 Å resolution (green; PDB ID: 8EVM) were aligned using TM-align (Zhang and Skolnick 2005) to 1.61 Å C_α RMSD. SP3x shares 94% sequence identity with the *de novo* chlorophyll dimer-binding protein SP3, but unlike SP3, SP3x can only bind one ZnPPaM molecule per protein dimer according to CD studies. While the SP3x design model largely matches the crystal structure, subtle discrepancies in the shape of the binding pocket and side chain rotameric states may contribute to the observed sub-stoichiometric ZnPPaM binding of SP3x. With the high-resolution SP3x structure in hand, we redesigned the binding cleft, allowing Rosetta FastRelax to select new binding site residues and make small adjustments to the ZnPPaM dimer geometry. We tested a new set of five mutants, one of which, SP3, exhibits high affinity assembly of a ZnPPaM dimer, as assayed by CD, UV/vis absorption, and fluorescence titrations (Supplementary Figures 4, 6, and 7). SP3 is designed to assemble a Chl dimer similar in structure to the P700 special pair of Photosystem I. Alignment of the SP3 design model to the P700 special pair from the crystal structure of Photosystem I (PDB ID: 1JB0) (Jordan et al. 2001) gives an RMSD of 0.68 Å across tetrapyrrole ring atoms. We grew green holo-state crystals of SP3 with ZnPPaM bound but were unable to solve the structure.

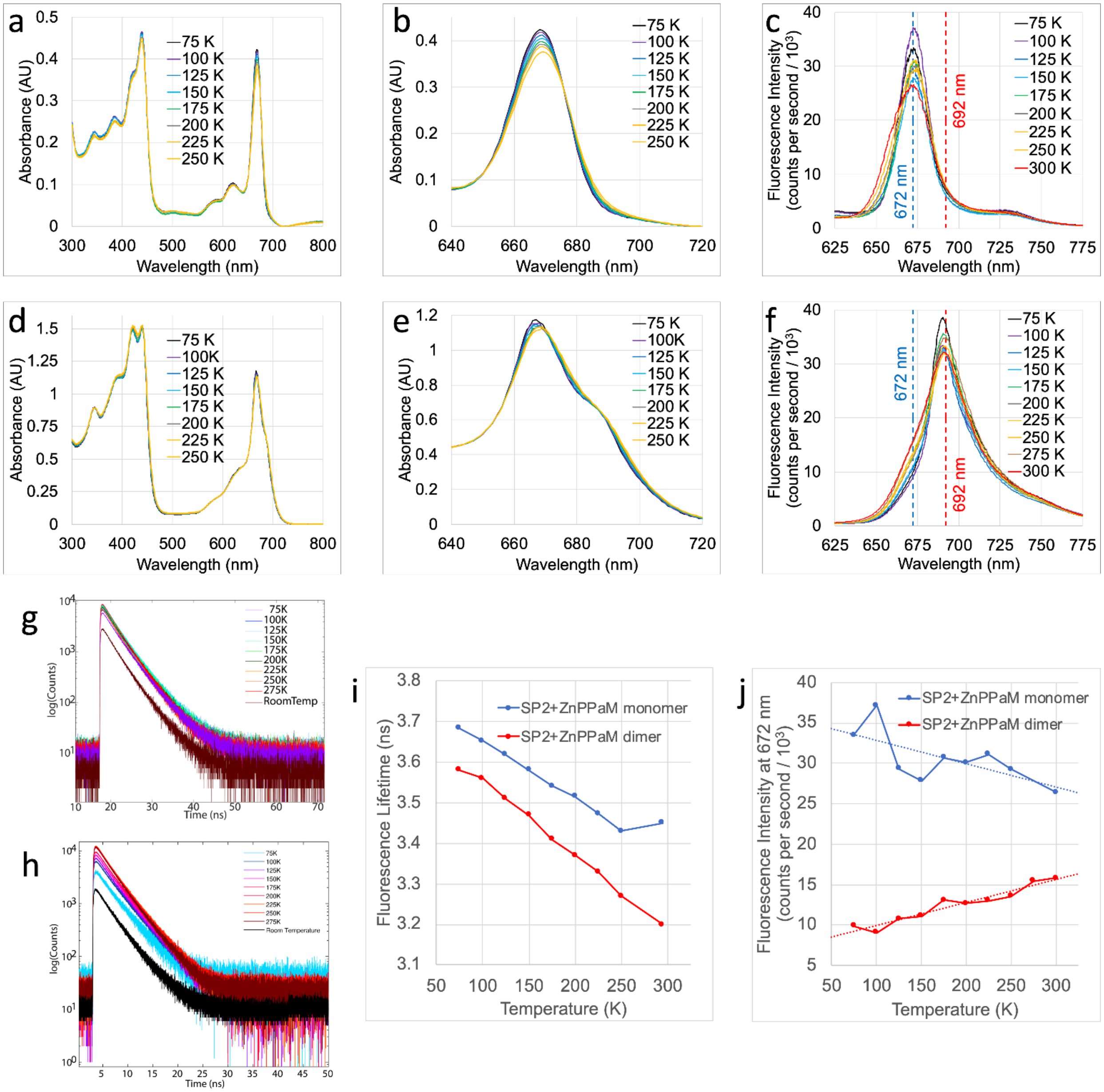

Supplementary Figure 9 | Temperature dependence of SP2-ZnPPaM absorbance and fluorescence in a sucrose/trehalose film.

(a) Full absorbance spectra, (b) absorbance in the Q_y region, and (c) fluorescence emission spectra of SP2-ZnPPaM monomer (prepared with 0.15 equivalents of ZnPPaM per SP2 protein monomer) at the indicated temperatures in sucrose/trehalose sugar matrix (Caram et al. 2016) (see Methods for details). Fluorescence excitation wavelength was 405 nm. (d) Full absorbance, (e) Q_y absorbance, and (f) fluorescence spectra of SP2-ZnPPaM dimer (prepared with 1.0 equivalent of ZnPPaM per SP2 protein monomer) in a sucrose/trehalose matrix. (g) Temperature dependent time correlated single photon counting (TCSPC) for SP2-ZnPPaM monomer and (h) SP2-ZnPPaM dimer. Excitation for TCSPC was 405 nm; the total fluorescent signal was collected after passing through a 420 nm long-pass filter to eliminate scattered laser light. (i) Fluorescence lifetimes of data in panels (g) and (h) fitted to mono-exponential decay and plotted against temperature. (j) Fluorescence intensities at 672 nm plotted against temperature for SP2-ZnPPaM monomer and dimer species from panels (c) and (f), respectively. Linear fits (dotted lines) indicate that temperature dependence of SP2-ZnPPaM fluorescence intensity has opposite sign for ZnPPaM monomer as compared to dimer.

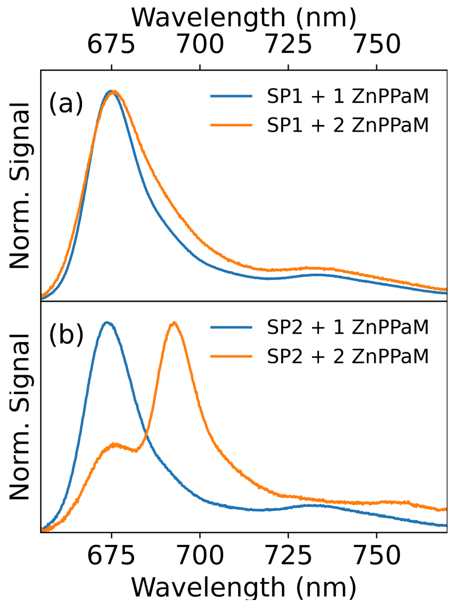

Supplementary Figure 10 | Experimental 77 K fluorescence spectra in 40% glycerol.
(a) SP1 and (b) SP2 homodimer proteins with 1 or 2 stoichiometric equivalents of ZnPPaM bound per complex. For 77 K measurements, protein samples in 150 mM NaCl and 10 mM Tris at pH 8 were diluted in a glycerol and water solution with the final glycerol concentration 40% v/v. The maximum OD for each sample in the Q_y region (near 670 nm) was adjusted to be ~0.06 (SP1 monomer: 0.07, SP1 dimer: 0.06, SP2 monomer: 0.08, and SP2 dimer: 0.06) and then diluted 20-fold using a 40% glycerol solution to avoid fluorescence reabsorption artifacts. The sample solution was placed in a transparent gelatin capsule (4.4 mm inner diameter) and plunge-frozen in liquid nitrogen to produce high-quality glass for spectroscopic measurements. Raw fluorescence spectra were corrected for (weak) wavelength-dependence of the detector response by calibration against a temperature-stabilized blackbody source (2796 K), and then residual light scattering from the glycerol matrix was eliminated by subtracting the measured spectrum of a gelatin capsule containing only 40% glycerol, scaled to match the intensity of the sample spectrum at 651.5 nm. The excitation source was a 450 nm Thorlabs laser diode (Thorlabs CPS450). The final laser power was ~567 µW, adjusted using an absorptive neutral density filter. The laser was focused on the sample with an approximate beam size of 2 mm x 1 mm. The appearance of a "monomer-like" band near 675 nm in the SP2 + 2 ZnPPaM spectrum likely indicates weak dissociation of the protein-ligand complex under these conditions.

Molecular dynamics and circular dichroism spectra calculations

We present here a more in-depth analysis of circular dichroism spectra calculations, together with molecular dynamics results.

The calculated spectra for SP1, using either the design model or the crystal structure, qualitatively match the experimental one. That is, the signs of the Cotton effects in the Q_y and Soret regions are both well reproduced (Supplementary Figure 8, cf. Figure 2 in the main manuscript). There is a slight discrepancy in the Q_y region, where calculations predict a conservative signal. The asymmetry in the experimental spectrum could be ascribed to a shielding effect from the protein environment (not explicitly considered in the calculations; see Methods for details). This shielding commonly decreases the effective electronic coupling (Curutchet et al. 2011; Adolphs and Renger 2006; Renger and Müh 2012) and, indeed, calculations where the couplings are scaled down reproduce the asymmetric feature in the Q_y region (Supplementary Figure 9). Without scaling, the calculated excitonic couplings between the Q_y states are 41 and 87 cm^{-1} for the design and crystal structures, respectively. A quantitative treatment of CD intensities likely requires a higher level of theory (Seibt, Lindorfer, and Renger 2022).

We also performed molecular dynamics simulations and tracked the chromophore dimer geometry, by monitoring the angle formed by the vectors that connect the NB and ND nitrogen atoms (these are approximately parallel to the Q_y transition dipole moments) (Georgakopoulou et al. 2002). The SP1 protein shows a very robust dimer geometry, with an angle between these vectors that is constant across 1.25 µs (Supplementary Figure 10) and is similar to the one from both the design and the crystal structure (137°). The distance between the metallic centers is stable as well, with an average value of 0.76 nm, similar to the 0.78 nm distance between Zn atoms in the crystal structure.

In the case of the SP2 crystal structure, calculations predict a strong excitonic coupling of 241 cm^{-1}, and an oscillator strength redistribution towards the high-energy exciton component (Supplementary Figure 11) that explains the low intensity red shoulder in the experimental absorption spectrum. The CD calculations on the SP2 crystal structure geometry reproduce the Cotton effect sign in the Q_y region, but not in the Soret (Supplementary Figure 8). Although the latter is more complicated due to the overlap of several transitions, the discrepancy could suggest slight rearrangements in the solution structure with respect to that of the crystal. On the other hand, the theoretical CD spectrum of the Chl dimer from the design model (which was shown to be incorrect by X-ray crystallography) had the sign of its Q_y Cotton effect inverted and had a different pattern in the Soret region (Supplementary Figure 8).

In addition, molecular dynamics simulations show a less stable dimer geometry for SP2 (Supplementary Figure 10), with a fluctuating relative orientation and an increased Zn – Zn distance consistent with the cavity expansion observed upon binding for this protein. An interesting observation is that simulations starting from the design structure undergo a change in dimer geometry, which brings the orientation and Zn – Zn distance closer to those of the crystal structure. This highlights the potential of molecular dynamics tools as a complement to protein design software (Barros et al. 2019).

Given the lack of a crystal structure for the SP3 protein in its holo state, we only performed CD calculations on the design model. As described in the main manuscript, the calculation reproduces the experimental features very well (Supplementary Figure 8), and yields an excitonic coupling value of 22 cm^{-1}. Considering the results for SP1 and SP2, the good spectral agreement can be taken as a confirmation that the dimer adopts the designed geometry.

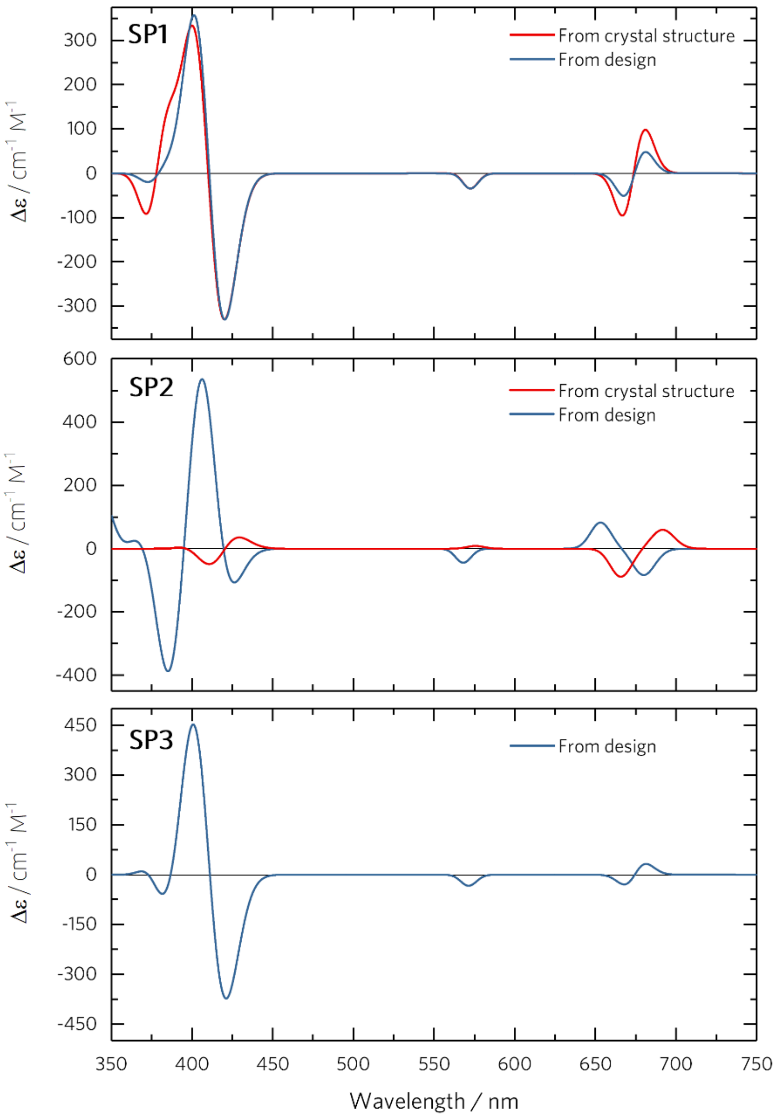

Supplementary Figure 11 | Calculated circular dichroism spectra, using either crystal structure or design geometries, for the SP1, SP2, and SP3 proteins with ZnPPaM dimer bound.

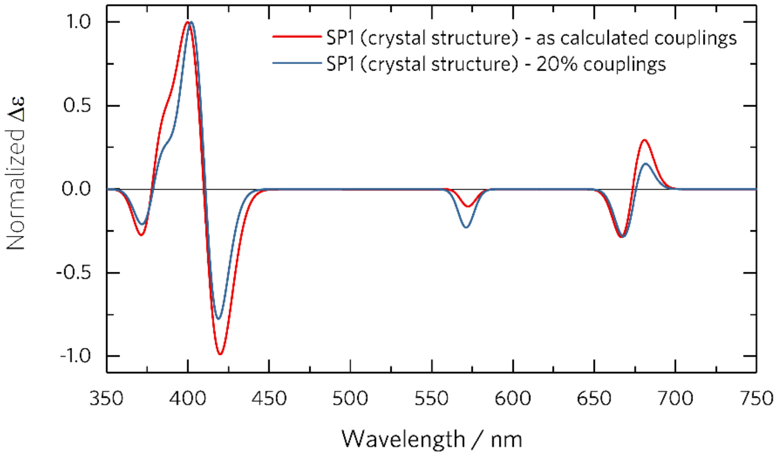

Supplementary Figure 12 | Calculated circular dichroism spectra for SP1-ZnPPaM using the crystal structure geometry (PDB ID: 7UNJ), and either full excitonic couplings, or scaled down to 20%.

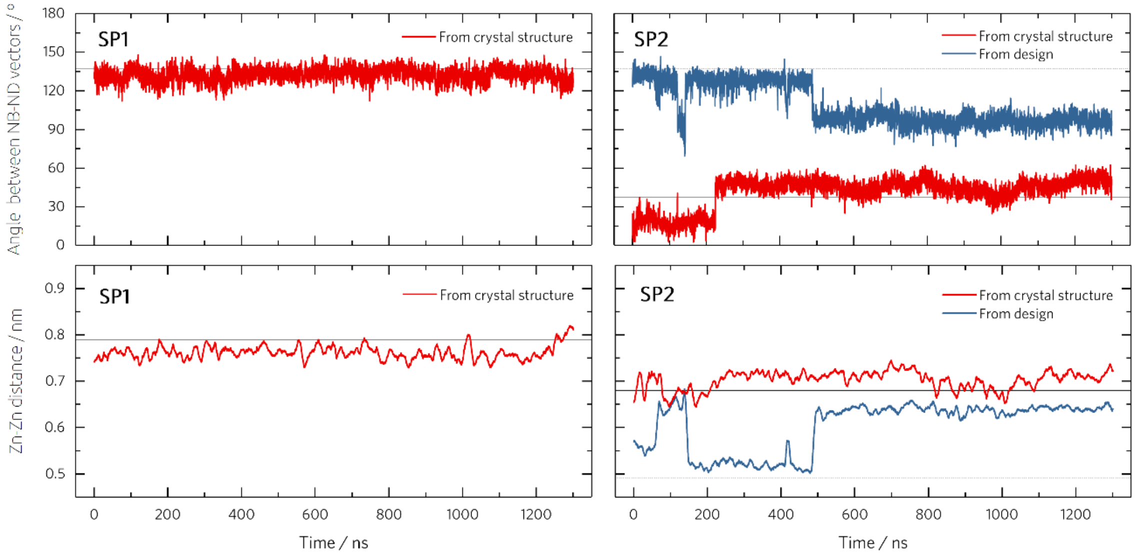

Supplementary Figure 13 | Structural parameters from molecular dynamics simulations on the SP1 (left) and SP2 (right) proteins, across 1.25 µs runs.
Top plots show the angle between the vectors that connect NB and ND nitrogen atoms in the chromophores, while bottom plots display the inter-chromophore distance expressed as the distance between their Zn atoms (50 points moving average). Horizontal solid lines show the values from the crystal structures, while dashed lines show the respective values from designs. Simulations were performed using either crystal structures or designed structures as initial points, as specified in the legends. Additional 0.5 µs replicas produced comparable results (data not shown).

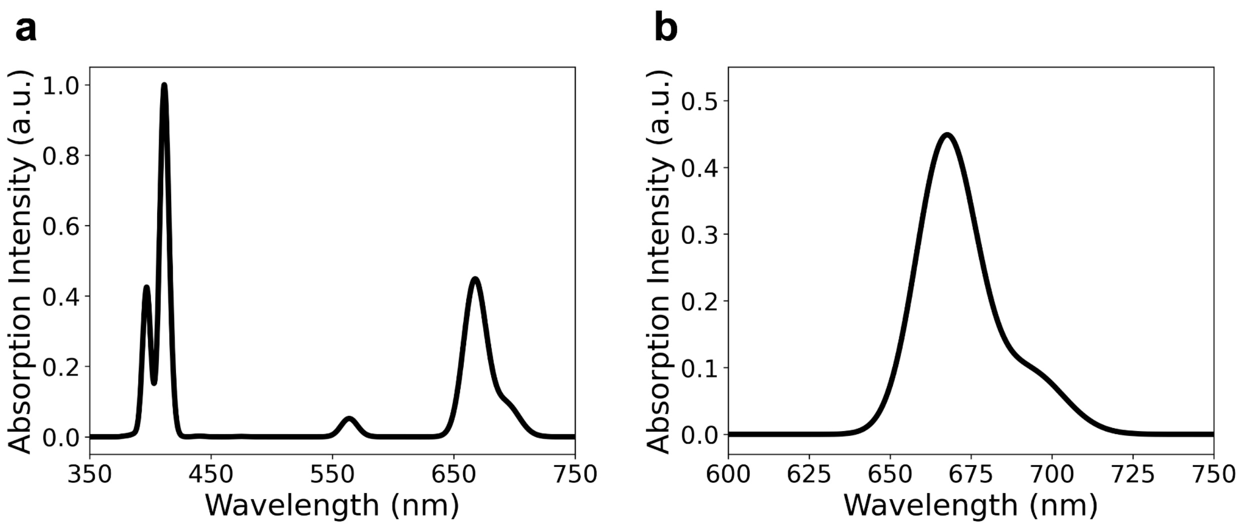

Supplementary Figure 14 | Calculated absorbance spectra for SP2-ZnPPaM using the crystal structure geometry.

(a) Calculated full UV/vis absorbance spectrum and (b) close-up of Q_y region, based on the SP2-ZnPPaM crystal structure (PDB ID: 7UNI). The calculated oscillator strength redistribution towards the high-energy exciton component in the Q_y region, which creates a stronger ~668 nm feature and a weaker ~690 nm band, is in agreement with the experimental SP2-ZnPPaM dimer absorbance spectrum as shown in the main text in Figure 4.

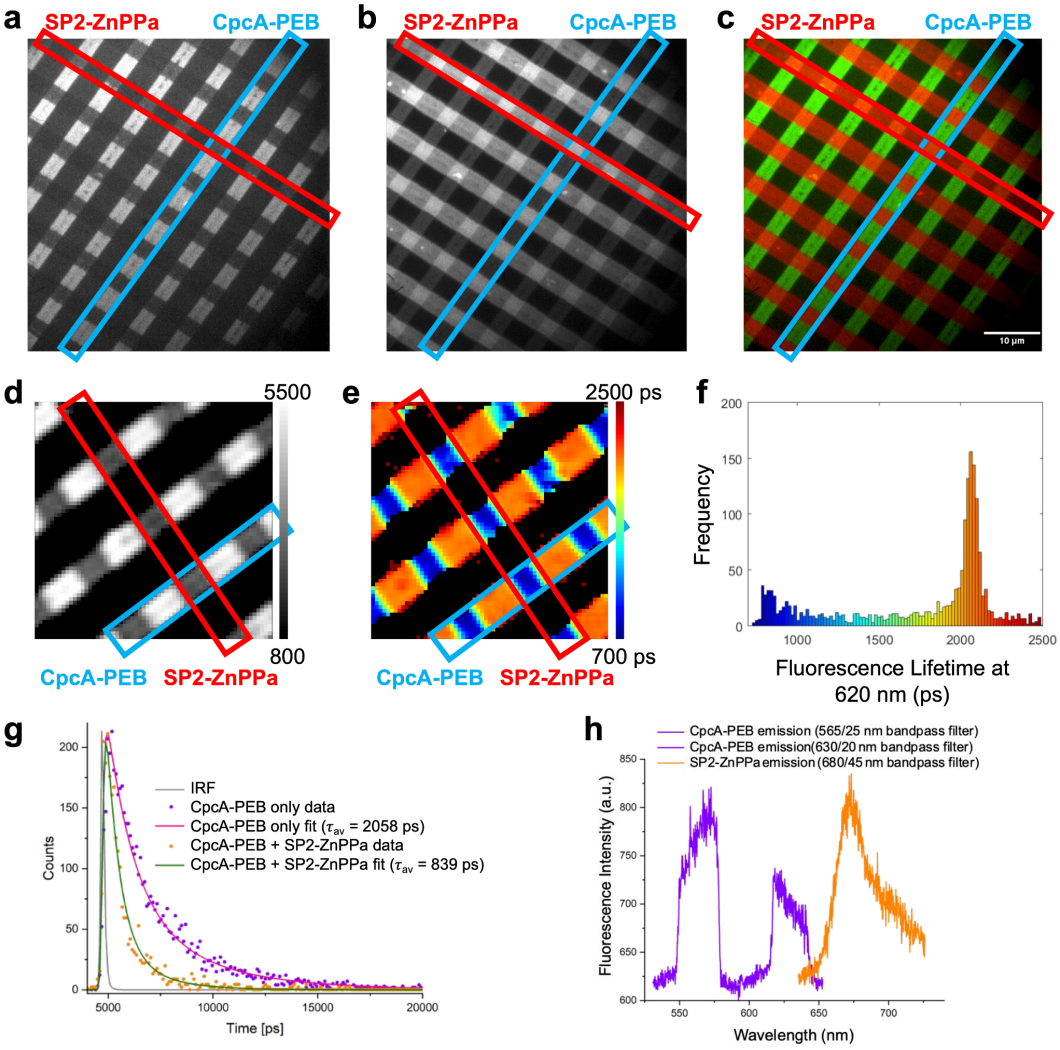

Supplementary Figure 15 | Energy transfer activity of designed special pair protein SP2 on a glass surface. Parallel lines (~5 µm-wide) of CpcA protein with bound phycoerythrobilin (PEB) were printed onto a glass surface by contact printing (Huang, Vasilev, and Hunter 2020). Lines of SP2-ZnPPa were printed perpendicular to and on top of the CpcA-PEB lines. (a) Image of proteins printed on functionalized glass showing total intensity of 620 nm emission. One of the CpcA-PEB lines is encapsulated by a blue box, and one line of SP2-ZnPPa is encapsulated by a red box. As expected, CpcA-PEB emits strongly at 620 nm, but SP2-ZnPPa does not. In locations where SP2-ZnPPa is printed on top of CpcA-PEB, 620 nm emission is significantly reduced. (b) The same substrate imaged at 680 nm, where SP2-ZnPPa emits, shows increased emission at 680 nm at locations where both CpcA-PEB and SP2-ZnPPa are present. (c) False color image showing 620 nm emission from panel (a) in green overlaid with 680 nm emission from panel (b) in red. (d) Total fluorescence emission at 620 nm similar to panel (a) with intensity indicated at right in arbitrary units. (e) Fluorescence lifetime map at 620 nm of same substrate region shown in panel (d) shows that SP2-ZnPPa shortens the fluorescence lifetime of CpcA-PEB. (f) A histogram of the amplitude-averaged lifetime showing a clear bimodal distribution, with peaks corresponding to the fluorescence lifetime values associated with the quenched and unquenched CpcA-PEB donor molecules. (g) Bi-exponentiial fits of representative fluorescence lifetime data. IRF is the instrument response function. Experimental data are shown as data points, and lines represent mono-exponential fits. Using E=1-(τ_{DA}/τ_D), where E is efficiency, τ_D is the fluorescence lifetime of the donor chromophore, and τ_{DA} is the lifetime of the donor in the presence of the acceptor, we find an energy transfer efficiency of 59%. (h) Fluorescence spectra of CpcA-PEB and SP2-ZnPPa when bandpass filters are applied as in data shown in panels (a) through (g).

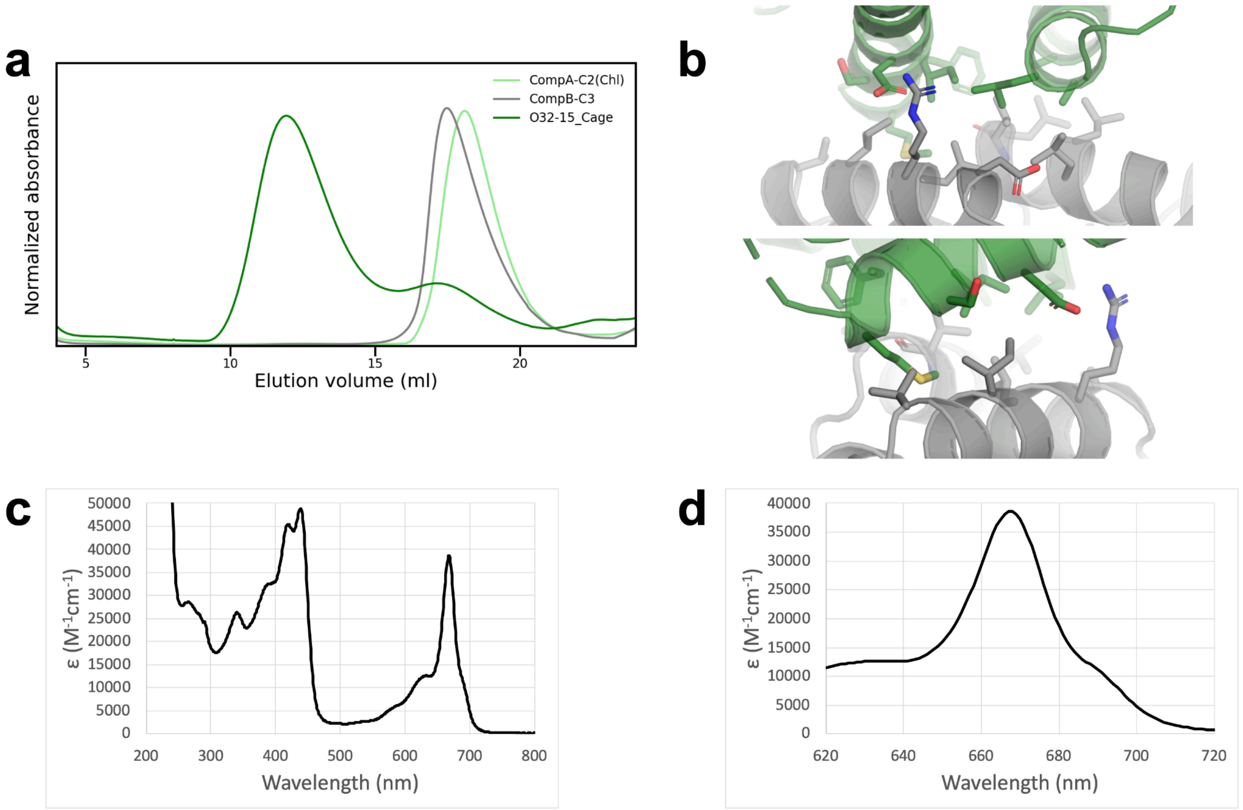

Supplementary Figure 16 | Design and characterization of chlorophyll-binding O32-15 nanocage.
(a) Size exclusion chromatography (SEC) chromatogram shows octahedral nanocage (green trace) elutes at the volume expected for the target 2-component cage (48-subunit) on a Superose 6 Increase 10/300 gel filtration column (Cytiva). The O32-15 cage was assembled in vitro by stoichiometric mixture of the individually produced and purified designed components: chlorophyll dimer (O32-15A, light green) and trimeric component (O32-15B, grey), respectively. (b) Two views of the design model of the cage interface within the two-component O32-15 chlorophyll binding cage. (c) UV/vis absorbance spectrum and (d) closeup of Q_y region of octahedral nanocage with chlorophyll bound after PD-10 column and sterile filtration. The spectrum includes a shoulder on the Q_y absorbance band at 692 nm, similar to SP2. The sample was prepared by adding a 1.8-fold excess of ZnPPaM (32 µM) to 2.5 mL of 17.6 µM nanocage (Chl binding site concentration) in 150 mM NaCl, 10 mM Tris at pH 8, and 0.3% w/v 3-((3-cholamidopropyl) dimethylammonio)-1-propanesulfonate (CHAPS) detergent. Sample was purified by PD-10 column to remove excess ZnPPaM and sterile filtered with a 0.22 µm syringe filter prior to collecting the absorbance spectrum shown.

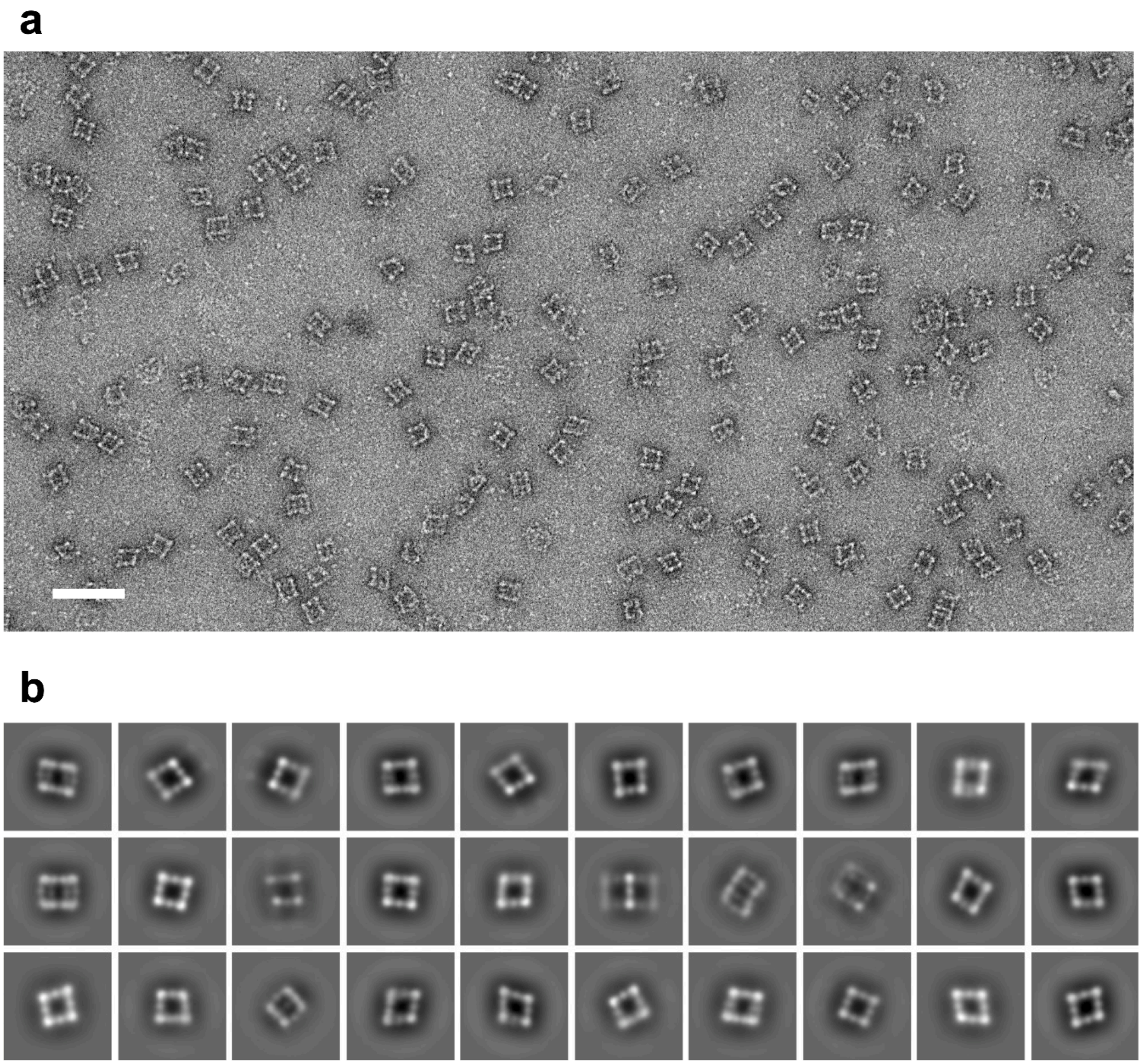

Supplementary Figure 17 | Negative-stain electron microscopy of purified O32-15 nanocage.
(a) Representative negative stain electron micrograph of 323 used in reconstruction (scale bar at bottom left, 100 nm). (b) 2D class averages of negative stain electron microscopy data created in CryoSparc (Punjani et al. 2017).

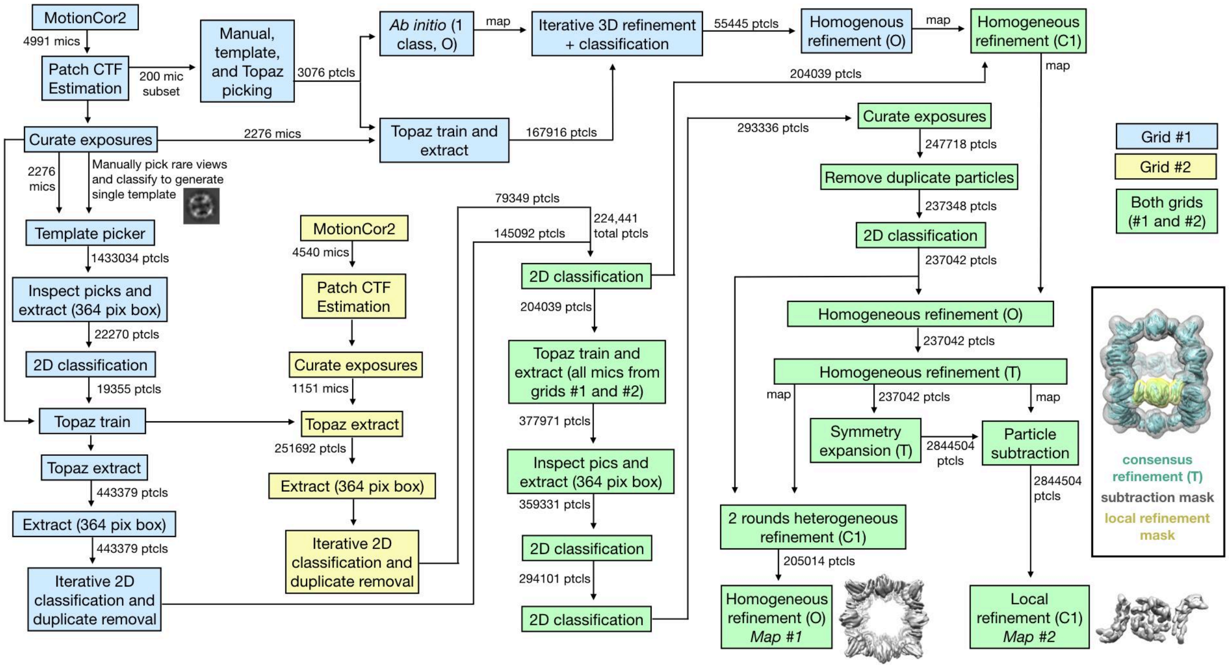

Supplementary Figure 18 | Cryo-EM data processing workflow for ZnPPaM-loaded nanocage.

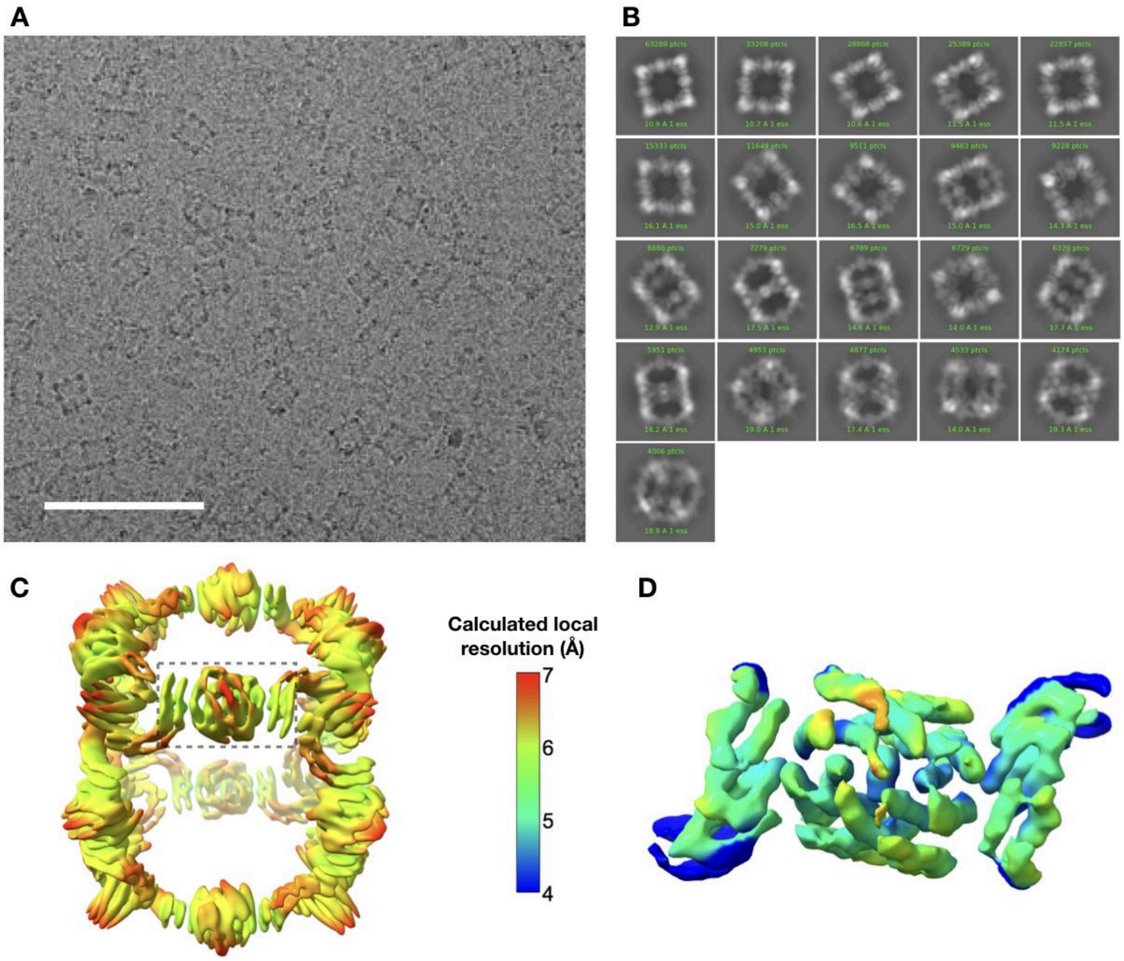

Supplementary Figure 19 | Cryo-EM of ZnPPaM-bound nanocage.
(A) Representative micrograph of 2,276 used in reconstruction; scale bar = 100 nm. (B) 2D averages of curated particle set (all classes containing at least 250 particles). (C) Cryo-EM maps of full nanocage and (D) ligand-binding region, colored by local resolution in Å, as calculated in Cryosparc. The region of the cage corresponding to the map in (D) is marked with a dashed rectangle in (C).

a

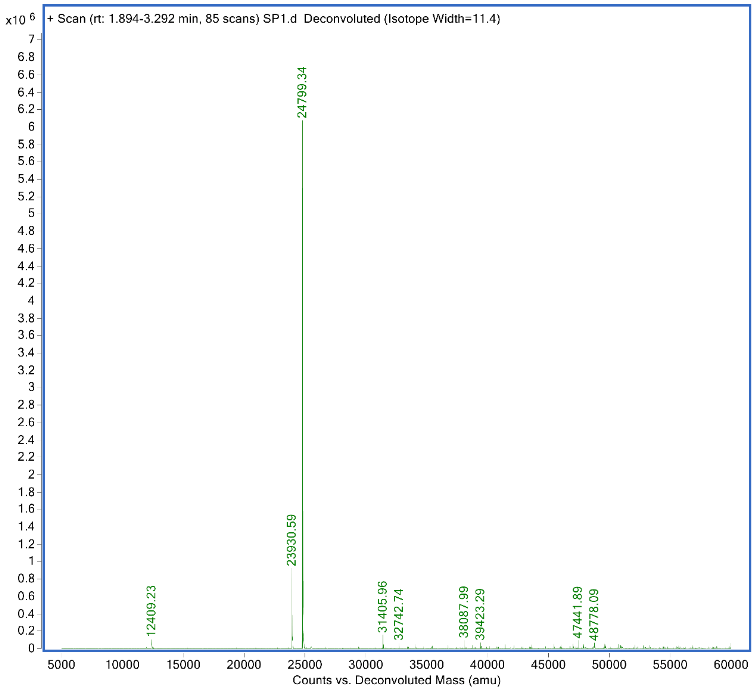

b

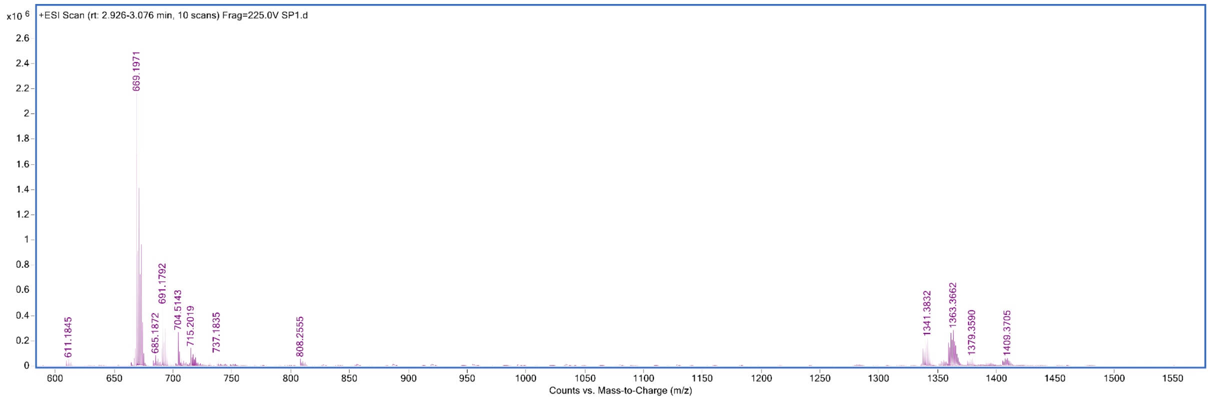

Supplementary Figure 20 | Reverse-phase liquid chromatography/mass spectrometry of SP1-ZnPPaM verifies molecular weights of protein and pigment.

(a) The dominant protein peak registers a molecular weight of 24799.3 Da; the expected protein molecular weight after cleavage by TEV protease is 24799.5 Da. (b) Mass spectrum of the same sample in the low-molecular weight range shows that ZnPPaM is present. The dominant peak at 669.2 Da is consistent with the expected molecular weight of 670.1 Da. Mass spectra were recorded after collecting CD and UV/vis spectra.

a

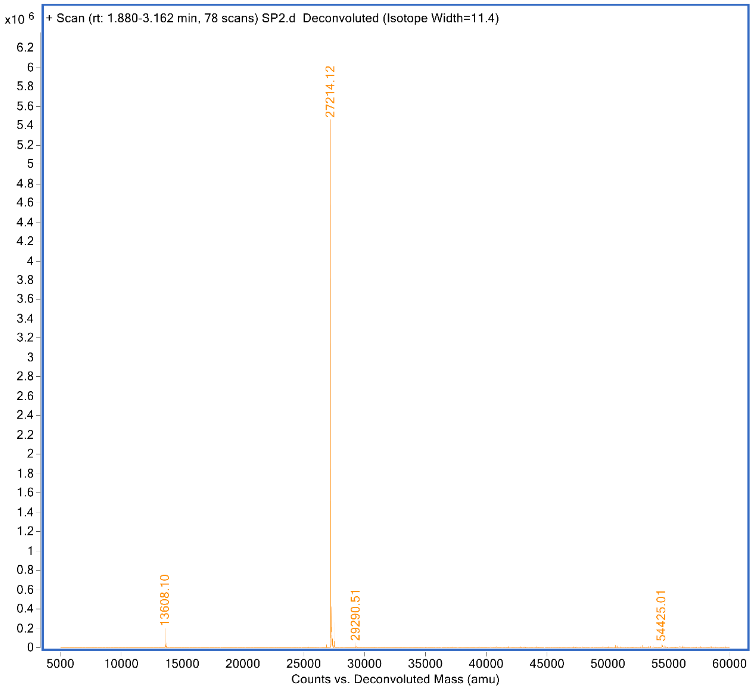

b

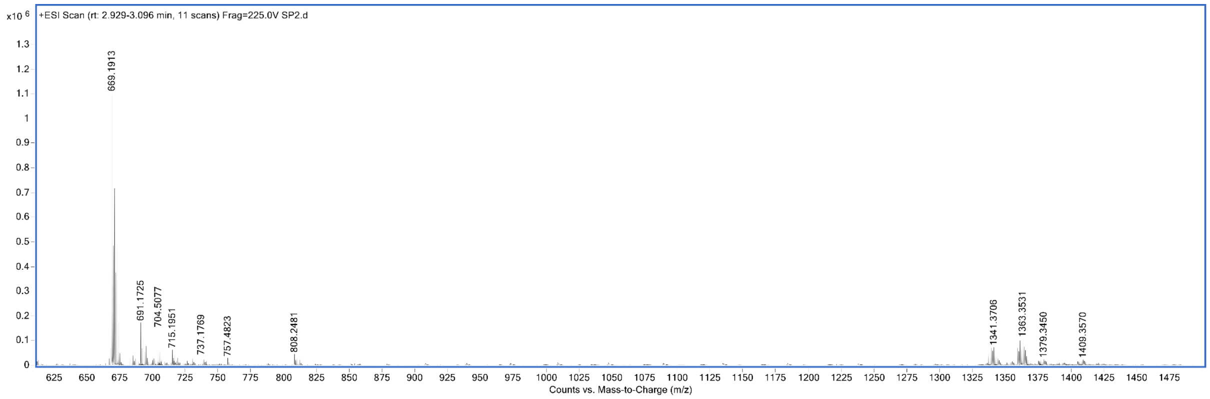

Supplementary Figure 21 | Reverse-phase liquid chromatography/mass spectrometry of SP2-ZnPPaM verifies molecular weights of protein and pigment.
(a) The dominant protein peak registers a molecular weight of 27214.1 Da; the expected protein molecular weight after cleavage by TEV protease is 27214.2 Da. (b) Mass spectrum of the same sample in the low-molecular weight range shows that ZnPPaM is present. The dominant peak at 669.2 Da is consistent with the expected molecular weight of 670.1 Da. Mass spectra were recorded after collecting CD and UV/vis spectra.

a

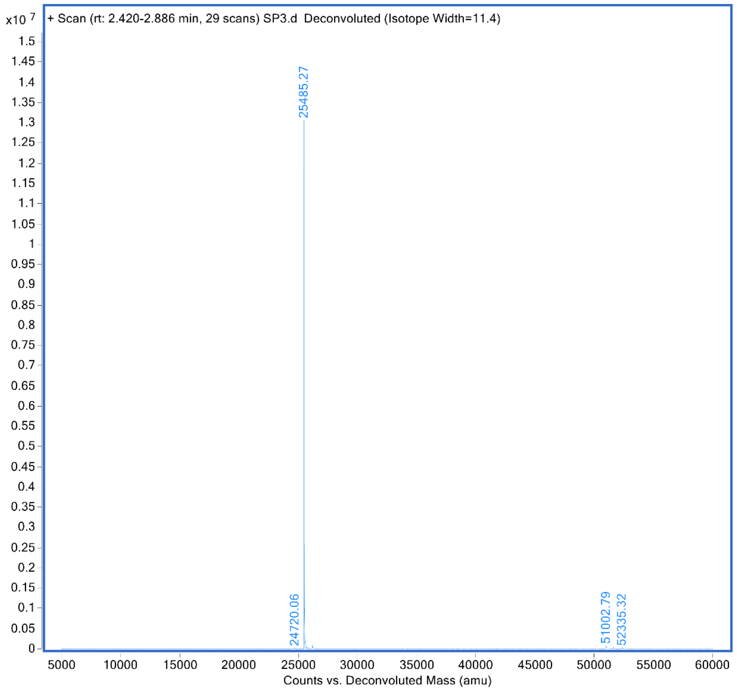

b

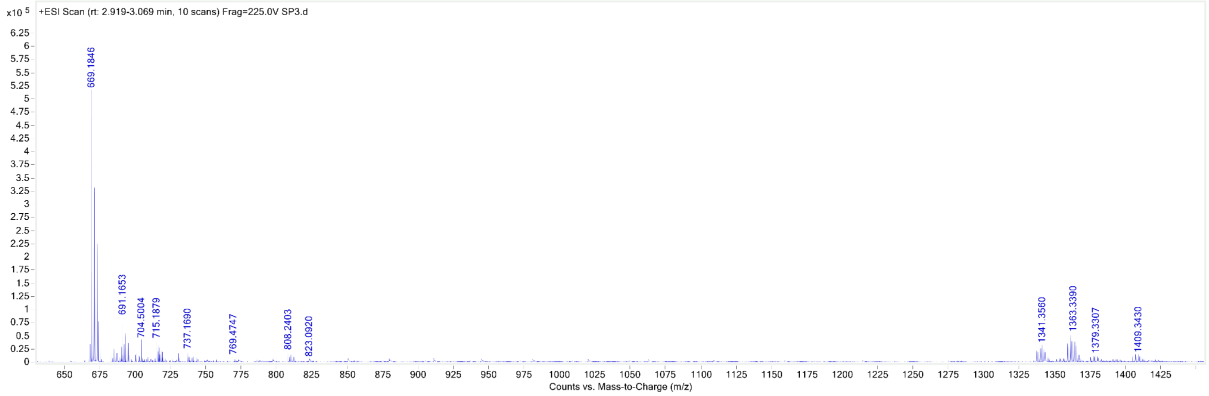

Supplementary Figure 22 | Reverse-phase liquid chromatography/mass spectrometry of SP3-ZnPPaM verifies molecular weights of protein and pigment.

(a) The dominant protein peak registers a molecular weight of 25485.3 Da; the expected protein molecular weight after cleavage by TEV protease is 25485.7 Da. (b) Mass spectrum of the same sample in the low-molecular weight range shows that ZnPPaM is present. The dominant peak at 669.2 Da is consistent with the expected molecular weight of 670.1 Da. Mass spectra were recorded after collecting CD and UV/vis spectra.

SP design	Amino acid sequence (after TEV protease digestion)
**SP1**	GGDSRELIARYQILLAELAAIRADIAAERTGDPYVRKLARELKRLAQEAAEEVKRDPSSSDVNMALLLILLMIELAVRALEAAERT<br>GDPEVRELAAELVWLAVEAAEEVQRNPSSSDVWLALHLIMLAIWAAVAALEAAERTGDPEVRELARELVRLAVEAAEEVQRNP<br>SSKEVYMALLLILIAILEAVLSLLRAERSGDPEKREKARERVREAVERAEEVQR
**SP2**	SGSGSSDEEFKFLATEAKMLITAAERLAGTDPELQEMVALIKKELEQAERTFRNGDKSEAQRQLEFVLTAARAVMNVAAAANAA<br>GTDPELIEMVLRILKQLKEAIRTFQNGDQEEAETQLRFVLRAAIAVAVVAAALVLAGTDPELQEMVKQILEELKQAIETFARGDKE<br>KALTQLLFVAWAAHAVAMIAAAANLAGTDPRLQQQVKEILEKLKEAIETFQKGDEEQAFRQLAEVLAEAALVALRAALTN
**SP3**	SGSSEELARESAEAAWRLAQAGTRLMLAWIRGDLKEIAEALIELARAVQELARVAKEYGNDELAKTAALLAAHVAMLAILVLIRAI<br>KEGDDEVRELAKTAIKLASTAAKIVLDALPTAEEVRQITLLAKLAEEAADKKNEDSALAVGIAAAAVLLALLALEAAQKAGIEEAEK<br>GARLLLKLAMDAARKKNPEEALAVFNAALDVSIALKLLQSAKRAGSEETRKLAEEMLRQALERARK
SP3x	SGSSEELARESAEAAWRLAQASTRATLAMIRGDLKELAEALIELARAVQELARVAKEYGNDELAKTAALLAAHVAMLAIWVLIRA<br>IKEGDDEVRELAKTAIKLASTAAKIVLDALPTAEEVRQITLLAKLAEEAADKKNEDSALAVGIAAIAVIIALWALEAAQKAGIEEAEK<br>GARLLLKLAMDAARKKNPEEALAVLNAALDVSIALQLLQSAKRAGSEETRKLAEEMLRQALERARKKN
JA	SGSGSWEEEAADKAIEIAEKVFRLKKSGTSEDEIAEEVAREISEVIRTLKESGSSEEVIALALLMIILGVVMALIANGVSIREQLDIS<br>ATILYEVAREQGNEEALEAIEEITRLAEEIKEEGGSILAAHILTMAGLLVAALPSEEAAEVAKEIAKAVKAAVEAEKDGKSDEAMLAL<br>IRLILAIMAALFNRSKYSLEMVRRIAKEAEQLAEDAREGK
JB	SGSTEELKKVLEDVRELAERAKESTDPEEALIIAILVIIRALAAVALDPSEEASTAVKIAIKIAEEVAKRVSDSELSASIALLIANLALVA<br>AKAAGDEELSKLALRLAKLAAELAKDALRIAEERGNAWQAAIAVGVVVMVATILLALAKQLGSEEALKEALEVAEEAARLAKRQL<br>ELAERVGDPIVALAAVGHVFMVAMLLAQIARESGSEEAKERAKRVAEEAEELAERVYELMRREGK
JC	SGSTEKLKKVLERVRELAERAKESTSPEEALIIALLVSSLAIAAVMLDPSEEASTAVKIAIKIAEEVAKRVSDSQLSASIALSIASLAL<br>VAAEAAGDEELSKLALRLAKLAAELAKDALRIAEERGNAIDAAIAVGIVVMVAAILLQLAKLLGSEEALKEALEVAEEAARLAKRVL<br>ELAERVGDPQVALAAVGHVQMVAILLAQIARESGSEEAKERAERVARESMELAERVLELMRREGKW
JD	SGSSDEEYARMSAELAEEAAKEALEQAKREGDEDARRVAEELEKQAEEARRKKDFDLANLVLMAAIILLLALIILELAKKAGNEQ<br>MEASGLSLLATAALLAAAGGENSEDARKALEAALRLAERATEVLEKIAKKGETALEKAIAAGTLVMLIQIIIQIAELLSKLGDTEEAK<br>KVLDIAIELIKRVTEILERIAKKADTDILAQAAAGHLIYLIQLLMQISILLRRLGNIDEAFKVLEEARELAERVRELLERIAKNSDK
JE	SGSGSSDELAALFLIISAKSQAWQAQSAAERTGDPRVRELARELKRLAQEAAEEVKRDPSSSEVNKNLQLISMAIALAVLALQL<br>ARKAGDEEARDIAREAVEVAVRAAQLILEGRDEQRAQTLFLIAHALLMAVMAARLGNSEVAELAVKVLRLAKELEKLLPSEIALFV<br>VLMIALAALSAAMAASGGQEDYARDALRRLEEAIREAEENRSKESLEKVIEEAIEAMLQANRAQTGG
JF	SGSGSSDELQARFLILSAKLQAEMAQIAAERTGDPRVRELARELKRLAQEAAEEVKRDPSSSEVNSNLALISMAIALAVLALLLA<br>RKAGDEEARDIAREAVEVAVRAAELILEGRDDQRAWTLWLIAHALLMAVYAARLGNSEVAELAVKVLRLAKELEKLLPSEEALFV<br>VSMIGLAALQAAMAAAGGQEDYARDALRRLEEAIREAEENRSKESLEKVEEEGIEAMEQALRAIAGG
JG	SGSGSSDELEARLLIISANLQAEMAQIAAERTGDPRVRELARELKRLAQEAAEEVKRDPSSSEVNQNLTLISMAIALAVLALLLA<br>RKAGDEEARDIAREAVEVAVRAAELILEGRDEQRAWTLFLIAHALLLAVLAARLGNSEVAELAVKVLRLAKELEKLLPSEIALFVV<br>SMIGMAALQAAMAASGGQEDYARDALRRLEEAIREAEENRSKESLEKVMEEAIRAYEQALRALAGG
JH	SGSSEELAREAREAAIRLAQASTKLMEAMIRGDLKELAEALIELARAVQELARVAKEYGNDELAKLAAFLAAHIAMLAIWVLILAIR<br>EGDDEVRELAKTAIKLASTAAKIVLDALPTAEEVRIITLLAKLAEEAADKKNEDSALAVLIAAMAVLIALAALEAAQRAGIEEAEKAA<br>RLLLKLAGEAARKKNPEEAKAVYNAALLVSIALLLLQKAKRAGSEETRRLAEEMLRQALERARKKN
D2K	SGSGSREKELKQLISNLQILLLILLSQDPNNELVRKLQEKIKEAEEQGDPRRLKLIWWILALINILQALEQEDPNNELVKRLKELIK<br>RALEEGDPRLLQLILLILHLIWLLQALEKEDPNNELVKRLKKLIEEALKEGDPRKLWQILALLLWIAQIQKLKKQDPNNELIKRAEET<br>LEQSLKT
D2L	SGSGSREKELKQLISNLQILLLILLSEDPNNELVRKLQEKIKEAEEQGDPRRLKLIWWILNLIQMLQQLEQEDPNNELVKRLKELIK<br>RALEEGDPRLLLLIHIILALIWLLQALEKEDPNNELVKRLKKLIEEALKEGDPRKLIQIALLLTWIATIQRLKKQDPNNELIKRAEETL<br>EQSLKT
D2M	SGSGSEEEQIRLAQLAIQIIQYQVEKNGDEQLLRLIQEITEQLQKEGDERLIAILSTMFGIIWILEQVEKNGDEELLRLIQEITEKLA<br>RQGDERLIQIGAAMLAIIIILEQVKKNGDEQLLRLIREITRKLAEEGDQRLIWILHAMMAIIMSSEELEKNGDEELLQKLRELLEKIA<br>KE
D2N	SGSGSEEEEIRLAQLTIQIIQYQVEKNGDEQLLRLIQEITEQLQKEGDERLIRILNAMFTIIWILEAVEKNGDEELLRLIQEITEKLAR<br>QGDERLIKILSAMAGIIWILEIVKKNGDEQLLRLIREITRKLAEEGDERLIQILANMHSIIISSEELEKNGDEELLQKLRELLEKIAKEL<br>EHHHHHH
D2O	SGSGSEEEEIRLAQLAIQIIQYQVEKNGDEQLLRLIQEITEQLQKEGDERLIRILLAMFAIILILEHVEKNGDEELLRLIQEITEKLAR<br>QGDERLIRILIAMAGIIWILELVKKNGDEQLLRLIREITRKLAEEGDERLIQILLAMHAIIISSEQLEKNGDEELLQKLRELLEKIAKE
D2P	SGSGSEEEEKRLIEKMSKFIWWFAQQLGDERILRAVEKLQQEIKENSPTPETFMTLINLLLSLLIWWAAQKLGDEKILEAVERLL<br>QQIEENSPTPETHMTLAALAQSLAIWQQAQKLGDEDILEAVERLLEEIQRNSPTPSTLIKLTWLLLLLNNWQELQKQGDEEQLR<br>SSEKAMKELKE

Supplementary Table 1 | Amino acid sequences of designed chlorophyll special pair proteins. Shown are the protein sequences after expression using a pET-29b(+) vector and cleavage of N-terminal MGHHHHHHGSGSGENLYFQ sequence by TEV protease.

D2Q	SGSGSEEEEKRLIEKMSKFIWWFAQQLGDERILRAVEKLQQEIKENSPTPETYMTLGNLLLSLLIWWAAQQLGDEKILEAVERLLQQIEENSPTPETHLTLAILMSALAIWQQAQKLGDEDILEAVERLLEEIQRNSPTPSTMTKLLWLALLLNNWEELQKQGDEEQLRSSEKAMKELKE
D2R	SGSGSEEEELKRLQTLIKYLQLLIQNAKWQNPDDELLRQVEELIEEMKKRLEENPNSQEDQALLKLLITIIALIIMILWLKSENPDDELLRQVEELIKEMIERLKENPNSQEDQALLQLLHAILGLILLIQQLKKQNPDDELLQQVEELIEEMIKRLKENPNSEKDQILLSFLALSLLSIFLIQTQKKQNPDDERLKQWEKQIKESIESSKE
D2S	SGSGSEEEELERLKEQLKIWQKLIKLILEQDPNDEQIRRILEQAEEIIQKLQEDPNNEELQRLIQLLFNLVAIIFSIKQILELDPNDEQIRRILEQAKEIIQKLQEDPNNEELFLLAHMLFALVAIILLIKLMLEQDPNDELIREILEQAKEIIQKLQEDPNNAELAMLIALLLSLVLWIWFMKFQLEQDPNDEEIRRQLEQLEERIRRTLE
D2T	SGSGSEEEELERLKEQLKIWQKLIKLILEQDPNDEQIRRILEQAEEIIQKLQEDPNNEELQRLIQLLFQLVLGIFIIKQILELDPNDEQIRRILEQAKEIIQKLQEDPNNEELYRLQGLLGMLVGIILLIKLMLEQDPNDELIREILEQAKEIIQKLQEDPNNQELLSLIHLLLALVLWIWFMKFQLEQDPNDEEIRRQLEQLEERIRRTLE
tj79C2-H62A	SGSGSRYDELNARLLILLAELAAERADIAAERTGDPRVRELARELKRLAQEAAEEVKRDPSSWSVLHALWLIVVAIEAAVRALEAAERTGDPEVRELARELVRLAVEAAEEVQRNPSSWDVFMALLLIVFAIEAAVRALEAAERTGDPEVRELARELVRLAVEAAEEVQRNPSSKEVDMALLLILIAILEAVLSLLRAERSGDPEKREKARERVREAVERAEEVQRDPSGW
tj79C2-H65A	SGSGSREDELLARYLILLAELAAEEADIAAERTGDPRVRELARELKRLAQEAAEEVKRDPSSSDVNHALHLIVLAILIAVEALEAAERTGDPEVRELARELVRLAVEAAEEVQRNPSSSDVLKALILILLAIIQAVKALEAAERTGDPEVRELARELVRLAVEAAEEVQRNPSSKEVTMALLLILIAILEAVLSLLRAERSGDPEKREKARERVREAVERAEEVQRDPSGW
tj79C2-H65B	SGSGSREDELFARYLILLAELAAERADIAAERTGDPRVRELARELKRLAQEAAEEVKRDPSSSDVNLALHLIVLAILAAVEALEAAERTGDPEVRELARELVRLAVEAAEEVQRNPSSSDVLKALMLIVLAILQAVQALEAAERTGDPEVRELARELVRLAVEAAEEVQRNPSSKEVYMALLLILIAILEAVLSLLRAERSGDPEKREKARERVREAVERAEEVQRDPSGW
tj79C2-H65C	SGSGSREDELIARYLILLAELAAERADIAAERTGDPRVRELARELKRLAQEAAEEVKRDPSSSDVFMALHLIVLAIQIAVKALEAAERTGDPEVRELARELVRLAVEAAEEVQRNPSSSDVFLALLLIVTAIMLAVEALEAAERTGDPEVRELARELVRLAVEAAEEVQRNPSSKEVLMALLLILIAILEAVLSLLRAERSGDPEKREKARERVREAVERAEEVQRDPSGW
tj79C2-H65D	SGSGSREDELQARLLILLIELAAERADIAAERTGDPRVRELARELKRLAQEAAEEVKRDPSSSDVWLALHLIVIAILIAVEALEAAERTGDPEVRELARELVRLAVEAAEEVQRNPSSSDVLKALMLILIAILLLLEALEAAERTGDPEVRELARELVRLAVEAAEEVQRNPSSKEVQMALLLILIAILEAILSLLRAERSGDPEKREKARERVREAVERAEEVQRDPSGW
tj79C2-H65E	SGSGSREDELWARLLILLIELAAERADIAAERTGDPRVRELARELKRLAQEAAEEVKRDPSSSDVLLALHLIVIAILIAVEALEAAERTGDPEVRELARELVRLAVEAAEEVQRNPSSSDVLIALMLILIAILLLLEALEAAERTGDPEVRELARELVRLAVEAAEEVQRNPSSKEVQMALLLILIAILEAILSLLRAERSGDPEKREKARERVREAVERAEEVQRDPSGW
tj79C2-H69A	SGSGSREDELQARLQLLAAELAAEEADIAAERTGDPRVRELARELKRLAQEAAEEVKRDPSSSDVNAALVLITHAIMLAVEALKAAERTGDPEVRELARELVRLAVEAAEEVQRNPSSSDVNKALFLIMMAIITAVTALEAAERTGDPEVRELARELVRLAVEAAEEVQRNPSSKEVDMALLLILIAILEAVLSLLRAERSGDPEKREKARERVREAVERAEEVQRDPSGW
tj79C2-H69B	SGSGSREDELHARYLILSAELAAERADIAAERTGDPRVRELARELKRLAQEAAEEVKRDPSSSDVNKALLLITHAILIAVAALEAAERTGDPEVRELARELVRLAVEAAEEVQRNPSSSDVNRALFLIMMAIMMAVKALEAAERTGDPEVRELARELVRLAVEAAEEVQRNPSSKEVDMALLLILIAILEAVLSLLRAERSGDPEKREKARERVREAVERAEEVQRDPSGW
tj79C2-H121A	SGSGSREDELIARYQILLAELAAERADIAAERTGDPRVRELARELKRLAQEAAEEVKRDPSSSDVNMALLLILLMIELAVRALEAAERTGDPEVRELARELVRLAVEAAEEVQRNPSSSDVWQALHLIMLAIWAAVAALEAAERTGDPEVRELARELVRLAVEAAEEVQRNPSSKEVYMALLLILIAILEAVLSLLRAERSGDPEKREKARERVREAVERAEEVQRDPSGW
tj79C2-H121B	SGSGSREDELLARLLLLLAELAAERADIAAERTGDPRVRELARELKRLAQEAAEEVKRDPSSSDVLMALLLIIMAIEAAVEALEAAERTGDPEVRELARELVRLAVEAAEEVQRNPSSSDVWWALHLILFAIQLAVRALEAAERTGDPEVRELARELVRLAVEAAEEVQRNPSSKEVDMALLLILIAILEAVLSLLRAERSGDPEKREKARERVREAVERAEEVQRDPSGW
tj79C2-H121C	SGSGSREDELLARLLILLAELAAERADIAAERTGDPRVRELARELKRLAQEAAEEVKRDPSSSDVLMLLLLIVLAIQAAVEALEAAERTGDPEVRELARELVRLAVEAAEEVQRNPSSSDVFMALHLILFAIQLAVQALEAAERTGDPEVRELARELVRLAVEAAEELQRNPSSKEVQMALLLILIAILEAVLSLLRAERSGDPEKREKARERVREAVERAEEVQRDPSGW
tj79C2-H125A	SGSGSREDELWARYTLLLAELAAERADIAAERTGDPRVRELARELKRLAQEAAEEVKRDPSSSDVYLALILIVMAIYMAVLALEAAERTGDPEVRELARELVRLAVEAAEEVQRNPSSSDVNKALLLIVHAIMAAVAALEAAERTGDPEVRELARELVRLAVEAAEEVQRNPSSKEVDMALLLIQIAILEAVLSLLRAERSGDPEKREKARERVREAVERAEEVQRDPSGW
tj79C2-H132A	SGSGSREDELRARLLILLAELAAETLDIIAERTGDPRVRELARELIRLLQEAAEEVKRDPSSSDVNEALKLIVEAIFMAIFILLFAEQTGDPEVRELARELVRLAVEAAEEVQRNPSSSDVNEALKLIIQAIILAVHALLAAEKTGDPEVRELARELVRLAVEAAEEVQRNPSSKEVDMALLLILIAILEAVLSLLRAQRSGDPEKREKARERVREAVERAEEVQRDPSGW
D13_H11A	SGSGSEEKEWQAMTLHLIFAIKILKEMLEEDPNDEEIKKILELLWQISKTQQDDPHNEEQKEWLMLLLLIIMALRLVQEMLKEDPNDEEIEKILELLKQILRTLREDPHNEEQKEWLILLLIIILLLDSVQEMLKEDPNDEEIEKILELLKQILRTLREDPHNEEQKQWLIQLINALILLLFIQEKLKEDPNDEEAEEELERLKEQLRRLRE
D13_H11B	SGSGSEEKEWQAMTAHLIYAIKILKEMLEEDPNDEEIKKILELLWQISKTQQDDPHNEEQKEWLILLLIIILALRLVQEMLKEDPNDEEIEKILELLKQILRTLREDPHNEEQKEWLSLLFIIIALLDSVQEMLKEDPNDEEIEKILELLKQILRTLREDPHNEEQKQWLIQLINALILLLFIQEKLKEDPNDEEAEEELERLKEQLRRLRE

Supplementary Table 1 (continued) | Amino acid sequences of designed chlorophyll special pair proteins.

D13_H11C	SGSGSEEKEWAIMTAHLIYAIKVLKEMLEEDPNDEEIKKILELLWQISKTQQDDPHNEEQKEWLMLLLGIILALRLVQEMLKEDPN<br>DEEIEKILELLKQILRTLREDPHNEEQKEWLALLVIIILLLDSVQEMLKEDPNDEEIEKILELLKQILRTLREDPHNEEQKQWLIQLIN<br>ALILLLFIQEKLKEDPNDEEAEEELERLKEQLRRLRE
D13_H60A	SGSGSEEKEWMLLTAMLIMAIKTLKEMLEEDPNDEEIKKILELLWQISKTQQDDPHNEEQKEWLHLLLIIIMALRLVQEMLKEDPN<br>DEEIEKILELLKQILRTLREDPHNEEQKEWLILLFIIIALLDSVQEMLKEDPNDEEIEKILELLKQILRTLREDPHNEEQKQWLIQLIN<br>ALILLLFIQEKLKEDPNDEEAEEELERLKEQLRRLRE
D13_H60B	SGSGSEEKEWQLLTWLLIMIIKTLKEMLEEDPNDEEIKKILELLWQISKTQQDDPHNEEQKEWLHLLAAIILALRLVQEMLKEDPN<br>DEEIEKILELLKQILRTLREDPHNEEQKAWLLLLVIIIALLDSVQEMLKEDPNDEEIEKILELLKQILRTLREDPHNEEQKQWLIQLIN<br>ALILLLFIQEKLKEDPNDEEAEEELERLKEQLRRLRE
JIa	SGSSEELARESAEAAWRLAQAGTRAALAAIRGDLKELAEALIELARAVQELARVAKEYGNDELAKTAALLAAHVALLAILVLIRAI<br>KEGDDEVRELAKTAIKLASTAAKIVLDALPTAEEVRQITLLAKLAEEAADKKNEDSALAVGIAAAAVLAALLALEAAQKAGIEEAEK<br>GARLLLKLAMDAARKKNPEEALAVLNAALDVSIALRLLQSAKRAGSEETRKLAEEMLRQALERARK
JIc	SGSSEELARESAEAAWRLAQAATRLVLALIRGDLKEVAEALIELARAVQELARVAKEYGNDELAKTAAWLAAHVAMLAIYVLIRAI<br>KEGDDEVRELAKTAIKLASTAAKIVLDALPTAEEVRQITLLAKLAEEAADKKNEDSALAVGIAAIAVLIALLALEAAQKAGIEEAEKG<br>ARLLLKLAMDAARKKNPEEALAVLNAALDVSIALRLLQSAKRAGSEETRKLAEEMLRQALERARK
JId	SGSSEELARESAEAAWRLAQAATRLVLAVIRGDLKEIAEALIELARAVQELARVAKEYGNDELAKTAAFLAAHVALLAIIVLIRAIKE<br>GDDEVRELAKTAIKLASTAAKIVLDALPTAEEVRQITLLAKLAEEAADKKNEDSALAVGIAAAAVLLALLALEAAQKAGIEEAEKGA<br>RLLLKLAMDAARKKNPEEALAVLNAALDVSIALRLLQSAKRAGSEETRKLAEEMLRQALERARK
JIe	SGSSEELARESAEAAWRLAQAATRIMLAVIRGDLKEIAEALIELARAVQELARVAKEYGNDELAKTAAFLAAHVALLAIIVLIRAIKE<br>GDDEVRELAKTAIKLASTAAKIVLDALPTAEEVRQITLLAKLAEEAADKKNEDSALAVGIAAMAVLIALLALEAAQKAGIEEAEKGA<br>RLLLKLAMDAARKKNPEEALAVFNAALDVSIALRLLQSAKRAGSEETRKLAEEMLRQALERARK
D_3_114_2	SGSGSSDEEFKWLATWAKMAITAAERAAGTDPELQEMVALIKKELEQAERTFRNGDKSEAWRQLVFVAIAAAAVLAVALAANA<br>AGTDPELQEMVDRILKQLKEAIRTFQNGDQEEAETQLFFVLLAAAAVAVVALALIWAGTDPELQEMVKQILEELKQAIETFARGD<br>KEKALTQLLFVLWAAHAVLWIAIAANTAGTDPRLQQQVKEILEKLKEAIETFQKGDEEQAFRQLAEVLAEAVLVALRAMKTN
D_3_114_3	SGSGSSDELFKWLATEAKMAITAAERLAGTDPELQEMVALIKKELEQAERTFRNGDKSEAMRQLLFVLMAANAVAAVAAAANM<br>AGTDPELQEMVDRILKQLKEAIRTFQNGDQEEAMTQLAFVLLAAAAVFVVAWALILAGTDPELQEMVKQILEELKQAIETFARG<br>DKEKALTQLLFVLWAAHAVAWIALAALLAGTDPRLLQQVKEILEKLKEAIETFQKGDEEQAFRQLAEVLAEAILVAIRAAKTN
D_3_114_4	SGSGSSDEEFKFLATMAKMAITAAERAAGTDPELQEMVALIKKELEQAERTFRNGDKSEAQRQLWFVWVAALAVAAVALAANA<br>AGTDPELQEMVDRILKQLKEAIRTFQNGDQEEAETQLRFVLMAAAAVAVVALARLWAGTDPELQEMVKQILEELKQAIETFARG<br>DKEKALTQLLFVLWAAHAVLWIAIAANLAGTDPRLQQQVKEILEKLKEAIETFQKGDEEQAFRQLAEVLAEAVLVALRARKTN
D_3_114_5	SGSGSSDFEFKMLATWAKMAITAAERAAGTDPELQEMVALIKKELEQAERTFRNGDKSEAMRQLEFVLTAANAVLAVALAANM<br>AGTDPELQEMVDRILKQLKEAIRTFQNGDQEEAEDQLLFVLAAATAVAVVAWAKILAGTDPELQEMVKQILEELKQAIETWARG<br>DKEKAATQLLFVMWAAHAVAAIALAANVAGTDPRLQQQVKEILEKLKEAIETFQKGDEEQAFRQLAEVLAEAMLVAIRALLTN

Supplementary Table 1 (continued) | Amino acid sequences of designed chlorophyll special pair proteins.

Protein name	R_G predicted from design model (Å)	R_G calculated from Guinier analysis of SAXS data (Å)
**SP1**	21.9	27.5
**SP2**	21.8	23.4
**SP3**	21.2	24.4
SP3x	21.4	24.4
JA	20.9	23.3
JB	21.9	24.0
JC	21.8	26.0
JD	22.3	28.2
JE	21.6	32.1
JF	21.6	32.6
JG	21.6	28.8
JH	21.5	26.7
D2T	22.4	25.5
tj79C2-H62A	22.2	27.4
tj79C2-H65B	22.4	26.3
tj79C2-H69A	22.6	26.3
tj79C2-H121A	22.4	26.0
tj79C2-H121B	22.5	25.3
tj79C2-H121C	22.4	25.4
D13_H60A	22.7	25.0

Supplementary Table 2 | Small angle X-ray scattering (SAXS) analysis of radii of gyration (R_G).
R_G values were calculated from SAXS profiles shown in Supplementary Figure 2 using Guinier Analysis in the program PRIMUS, part of the ATSAS software package (Manalastas-Cantos et al. 2021). Expected R_G values based on apo-states of design models and data fitting were calculated using the FoXS server (Schneidman-Duhovny et al. 2013, 2016). The systematic underprediction of R_G values (including for proteins with solved X-ray crystal structures) is likely the result of SAXS detection of dense hydration shells around the highly charged SP proteins that was not accounted for in design models (Kim et al. 2016; Svergun et al. 1998).

Sample	Absorbance Maximum (ε, λ)	CD minimum or maximum (Δε, λ)	Δε/ε Ratio
ZnPPaM in DEF	78,000 $M^{-1}cm^{-1}$, 658 nm	-16.2 $M^{-1}cm^{-1}$, 657 nm	-2.1×10^{-4}
ZnPPaM in DMSO	76,000 $M^{-1}cm^{-1}$, 660 nm	-17.8 $M^{-1}cm^{-1}$, 663 nm	-2.3×10^{-4}
ZnPPaM in MeOH	71,000 $M^{-1}cm^{-1}$, 657 nm	-14.7 $M^{-1}cm^{-1}$, 659 nm	-2.1×10^{-4}
SP1-ZnPPaM	49,000 $M^{-1}cm^{-1}$, 667 nm	-10.4 $M^{-1}cm^{-1}$, 661 nm	-2.1×10^{-4}
		4.0 $M^{-1}cm^{-1}$, 684 nm	0.8×10^{-4}
SP2-ZnPPaM	38,200 $M^{-1}cm^{-1}$, 668 nm	-16.5 $M^{-1}cm^{-1}$, 674 nm	-4.3×10^{-4}
		12.7 $M^{-1}cm^{-1}$, 698 nm	3.3×10^{-4}
SP3-ZnPPaM	39,000 $M^{-1}cm^{-1}$, 666 nm	-16.7 $M^{-1}cm^{-1}$, 664 nm	-4.3×10^{-4}
		16.9 $M^{-1}cm^{-1}$, 694 nm	4.3×10^{-4}

Supplementary Table 3 | ZnPPaM CD signal intensities in SP proteins compared to organic solvent controls.
The Δε/ε ratio gives an indication of the CD signal intensity relative to absorbance. In organic solvents, ZnPPaM has a non-conservative negative CD transition in the Q_y region with a Δε/ε ratio of -2.1×10^{-4} to -2.3×10^{-4}. In the weakly coupled ZnPPaM dimer of SP1, the transition is similarly asymmetric with a comparable Δε/ε ratio, but a smaller positive peak is also present at 684 nm. In contrast, the conservative Cotton effects of ZnPPaM bound to either SP2 or SP3 have close to double the Δε/ε ratio, consistent with stronger excitonic coupling between the chromophores. See Figure 2 in the main text and Supplementary Figure 3 for spectra.

	SP1-ZnPPaM (PDB ID: 7UNJ)	SP2 (apo-state) (PDB ID: 7UNH)	SP2-ZnPPaM (PDB ID: 7UNI)	SP3x (apo-state) (PDB ID: 8EVM)
Well solution	32% (w/v) PEG-3350, 200 mM lithium sulfate, 100 mM BisTris pH 6.5	24% (w/v) PEG-3350, 140 mM KCl	30% (w/v) PEG-3350, 100 mM ammonium sulfate	2.4 M sodium malonate dibasic monohydrate, pH 7.0
**Data Collection**				
Space Group	$P4_1$	$P2_1$	P1	$P3_1$ 2 1
Unit Cell Dimensions	a=b=52.35 Å, c=173.72 Å, a=b=c=90°	a=54.13 Å, b=76.1 Å, c=63.23 Å, b=99.06°, a=c=90°	a=52.8 Å, b=54.9 Å, c=89.3 Å, a=87.83°, b=84.06°, c=69.45°	a=b=88.732 Å, c=149.484 Å, a=b=90°, c=120°
Resolution	36.2 - 2.0 Å (2.07 - 2.0)	28.89 - 2.4 Å (2.46-2.4)	49.26 - 2.07 Å (2.13 - 2.07)	44.37 - 3.05 Å (3.16 - 3.05)
Reflections	31367	19927	31294	13483 (1311)
Completeness (%)	99.8 (99.5)	98.2 (98.2)	92.3 (60.2)	99.42 (96.27)
Redundancy	13.0 (13.2)	48.7 (49.7)	3.5 (3.4)	10.8 (10.7)
I/s(I)	32.4 (5.4)	50.1 (22.7)	7.5 (0.9)	11.54 (0.62)
R_{merge}	0.066 (0.429)	0.082 (0.213)	0.067 (1.297)	0.1943 (5.037)
R_{pim}	0.019 (0.121)	0.012 (0.030)	0.043 (0.834)	0.0612 (1.595)
CC1/2	0.999 (0.967)	1.01 (0.84)	0.98 (0.97)	0.999 (0.248)
**Refinement**				
Resolution	36.2 - 2.0 Å	28.89 - 2.4 Å	49.26 - 2.5 Å	44.37 - 3.05 Å
R_{work} / R_{free}	0.196 (0.219) / 0.237 (0.291)	0.209 (0.223) / 0.263 (0.322)	0.201 (0.258) / 0.251 (0.321)	0.2509 (0.3917) / 0.3017 (0.4188)
Rmsd bonds	0.006 Å	0.002 Å	0.007 Å	0.003 Å
Rmsd angles	0.76°	0.37°	0.86°	0.55°
Wilson B-factor	38.31	22.70	52.90	112.91
Number of protein atoms	3203	3656	6684	3539
Number of ligand atoms	107	8	206	0
Number of water atoms	65	189	9	0
Ramachandran Distribution (Favored%/ Allowed%/ Outlier%)	99.54 / 0.23 / 0.23	99.38 / 0.62 / 0.0	97.7 / 2.09 / 0.21	98.08 / 1.92 / 0.0

Supplementary Table 4 | Summary of X-ray crystallographic data collection and refinement statistics.

Microscope	Titan Krios
Voltage (kV)	300
Exposure navigation	Image shift
Nominal magnification	64000X
Detector	Gatan K3
Pixel size (Å/pix)	1.076
No. of frames	40
Dose rate (e-/Å/s)	24.99
Exposure per frame (e-/Å^2)	1.25
Total dose (e-/Å^2)	49.99
Exposure time (s)	2.0
Defocus range (μm)	0.3 - 2.5
Number of grids imaged	2
Total number of micrographs	9531

Supplementary Table 5 | CryoEM data acquisition.

Image Processing	**Full nanocage (*Map #1)***	***ZnPPaM-binding region (Map #2)***
Software used	Cryosparc v.3	Cryosparc v.3
No. of extracted particles	359331	
No. of final particles	205014	237042
No. of refined particles	205014	2844504 (expanded w/T symmetry)
Box size (pixels)	364	364
Symmetry imposed in final refinement	O	None (C1)
Global map resolution (FSC 0.143 unmasked)	> 6Å	> 5Å
Map sharpening B factor ($Å^2$)	none	none
EMDB ID	EMD-40208	EMD-40209
**Supplementary Table 6 | CryoEM data processing.**		

Map CC (mask)	0.62
Map CC (volume)	0.63
Map CC (peaks)	0.55
R.m.s. deviations (bonds)	0.002
R.m.s. deviations (angles)	0.456
Ramachandran plot values (%)	
outliers	0
allowed	2.14
favored	97.86
C-beta deviations (%)	0
CaBLAM outliers (%)	1.09
Overall score (Molprobity (Williams et al. 2018))	1.47
Clashscore	8.05
PDB ID	8GLT

Supplementary Table 7 | Statistics for nanocage polyalanine model fitted and refined to cryo-EM map (EMD-40208).

Supplemental References

Adolphs, Julia, and Thomas Renger. 2006. "How Proteins Trigger Excitation Energy Transfer in the FMO Complex of Green Sulfur Bacteria." *Biophysical Journal* 91 (8): 2778–97.

Barros, Emilia P., Jamie M. Schiffer, Anastassia Vorobieva, Jiayi Dou, David Baker, and Rommie E. Amaro. 2019. "Improving the Efficiency of Ligand-Binding Protein Design with Molecular Dynamics Simulations." *Journal of Chemical Theory and Computation* 15 (10): 5703–15.

Caram, Justin R., Sandra Doria, Dörthe M. Eisele, Francesca S. Freyria, Timothy S. Sinclair, Patrick Rebentrost, Seth Lloyd, and Moungi G. Bawendi. 2016. "Room-Temperature Micron-Scale Exciton Migration in a Stabilized Emissive Molecular Aggregate." *Nano Letters* 16 (11): 6808–15.

Curutchet, Carles, Jacob Kongsted, Aurora Muñoz-Losa, Hoda Hossein-Nejad, Gregory D. Scholes, and Benedetta Mennucci. 2011. "Photosynthetic Light-Harvesting Is Tuned by the Heterogeneous Polarizable Environment of the Protein." *Journal of the American Chemical Society* 133 (9): 3078–84.

Dyer, Kevin N., Michal Hammel, Robert P. Rambo, Susan E. Tsutakawa, Ivan Rodic, Scott Classen, John A. Tainer, and Greg L. Hura. 2014. "High-Throughput SAXS for the Characterization of Biomolecules in Solution: A Practical Approach." *Methods in Molecular Biology* 1091: 245–58.

Georgakopoulou, Sofia, Raoul N. Frese, Evelyn Johnson, Corline Koolhaas, Richard J. Cogdell, Rienk van Grondelle, and Gert van der Zwan. 2002. "Absorption and CD Spectroscopy and Modeling of Various LH2 Complexes from Purple Bacteria." *Biophysical Journal* 82 (4): 2184–97.

Huang, Xia, Cvetelin Vasilev, and C. Neil Hunter. 2020. "Excitation Energy Transfer between Monomolecular Layers of Light Harvesting LH2 and LH1-Reaction Centre Complexes Printed on a Glass Substrate." *Lab on a Chip* 20 (14): 2529–38.

Jordan, P., P. Fromme, H. T. Witt, O. Klukas, W. Saenger, and N. Krauss. 2001. "Three-Dimensional Structure of Cyanobacterial Photosystem I at 2.5 A Resolution." *Nature* 411 (6840): 909–17.

Kim, Henry S., Anne Martel, Eric Girard, Martine Moulin, Michael Härtlein, Dominique Madern, Martin Blackledge, Bruno Franzetti, and Frank Gabel. 2016. "SAXS/SANS on Supercharged Proteins Reveals Residue-Specific Modifications of the Hydration Shell." *Biophysical Journal* 110 (10): 2185–94.

Manalastas-Cantos, Karen, Petr V. Konarev, Nelly R. Hajizadeh, Alexey G. Kikhney, Maxim V. Petoukhov, Dmitry S. Molodenskiy, Alejandro Panjkovich, et al. 2021. "ATSAS 3.0: Expanded Functionality and New Tools for Small-Angle Scattering Data Analysis." *Journal of Applied Crystallography* 54 (Pt 1): 343–55.

Punjani, Ali, John L. Rubinstein, David J. Fleet, and Marcus A. Brubaker. 2017. "cryoSPARC: Algorithms for Rapid Unsupervised Cryo-EM Structure Determination." *Nature Methods* 14 (3): 290–96.

Renger, Thomas, and Frank Müh. 2012. "Theory of Excitonic Couplings in Dielectric Media : Foundation of Poisson-TrEsp Method and Application to Photosystem I Trimers." *Photosynthesis Research* 111 (1-2): 47–52.

Schneidman-Duhovny, Dina, Michal Hammel, John A. Tainer, and Andrej Sali. 2013. "Accurate SAXS Profile Computation and Its Assessment by Contrast Variation Experiments." *Biophysical Journal* 105 (4): 962–74.

———. 2016. "FoXS, FoXSDock and MultiFoXS: Single-State and Multi-State Structural Modeling of Proteins and Their Complexes Based on SAXS Profiles." *Nucleic Acids Research* 44 (W1): W424–29.

Seibt, Joachim, Dominik Lindorfer, and Thomas Renger. 2022. "Signatures of Intramolecular Vibrational and Vibronic Q_x–$Q_y$coupling Effects in Absorption and CD Spectra of Chlorophyll Dimers." *Photosynthesis Research*, August. https://doi.org/10.1007/s11120-022-00946-3.

Svergun, D. I., S. Richard, M. H. Koch, Z. Sayers, S. Kuprin, and G. Zaccai. 1998. "Protein Hydration in Solution: Experimental Observation by X-Ray and Neutron Scattering." *Proceedings of the National Academy of Sciences of the United States of America* 95 (5): 2267–72.

Weber, G., and F. W. J. Teale. 1957. "Determination of the Absolute Quantum Yield of Fluorescent Solutions." *Transactions of the Faraday Society* 53 (0): 646–55.

Williams, Christopher J., Jeffrey J. Headd, Nigel W. Moriarty, Michael G. Prisant, Lizbeth L. Videau, Lindsay N. Deis, Vishal Verma, et al. 2018. "MolProbity: More and Better Reference Data for Improved All-Atom Structure Validation." *Protein Science: A Publication of the Protein Society* 27 (1): 293–315.

Zhang, Yang, and Jeffrey Skolnick. 2005. "TM-Align: A Protein Structure Alignment Algorithm Based on the TM-Score." *Nucleic Acids Research* 33 (7): 2302–9.
