# Supplementary material for: De novo design of proteins housing excitonically coupled chlorophyll special pairs
Source: Nat Chem Biol. 2024 Jun 3;20(7):906–15. doi: 10.1038/s41589-024-01626-0 (PMC11213709; doi:10.1038/s41589-024-01626-0)
Supplement: Supplementary file 2 — Reporting Summary [file 41589_2024_1626_MOESM2_ESM.pdf]

nature portfolio

Corresponding author(s): Nathan M. Ennist
David Baker

Last updated by author(s): Jan 25, 2024

Reporting Summary

Nature Portfolio wishes to improve the reproducibility of the work that we publish. This form provides structure for consistency and transparency in reporting. For further information on Nature Portfolio policies, see our Editorial Policies and the Editorial Policy Checklist.

Statistics

For all statistical analyses, confirm that the following items are present in the figure legend, table legend, main text, or Methods section.

n/a	Confirmed	
☐	☒	The exact sample size (*n*) for each experimental group/condition, given as a discrete number and unit of measurement
☐	☒	A statement on whether measurements were taken from distinct samples or whether the same sample was measured repeatedly
☒	☐	The statistical test(s) used AND whether they are one- or two-sided<br>*Only common tests should be described solely by name; describe more complex techniques in the Methods section.*
☒	☐	A description of all covariates tested
☐	☒	A description of any assumptions or corrections, such as tests of normality and adjustment for multiple comparisons
☐	☒	A full description of the statistical parameters including central tendency (e.g. means) or other basic estimates (e.g. regression coefficient) AND variation (e.g. standard deviation) or associated estimates of uncertainty (e.g. confidence intervals)
☒	☐	For null hypothesis testing, the test statistic (e.g. *F*, *t*, *r*) with confidence intervals, effect sizes, degrees of freedom and *P* value noted<br>*Give P values as exact values whenever suitable.*
☒	☐	For Bayesian analysis, information on the choice of priors and Markov chain Monte Carlo settings
☒	☐	For hierarchical and complex designs, identification of the appropriate level for tests and full reporting of outcomes
☒	☐	Estimates of effect sizes (e.g. Cohen's *d*, Pearson's *r*), indicating how they were calculated

Our web collection on statistics for biologists contains articles on many of the points above.

Software and code

Policy information about availability of computer code

Data collection	The Rosetta macromolecular modeling suite (https://www.rosettacommons.org) is freely available to academic and non-commercial users. Commercial licenses for the suite are available through the University of Washington Technology Transfer Office. A python package and example scripts for Chl docking can be found here: https://github.com/atommoyer/stapler. RPXDock, version 2 is available here: https://github.com/willsheffler/rpxdock.
Data analysis	Software packages used for data analysis are described in Methods in detail. Mass spectra were deconvoluted in Bioconfirm using a total entropy algorithm. SAXS data were processed in SAXS Frameslice version 1.4.13 and compared to design models using FoXS server (https://modbase.compbio.ucsf.edu/foxs/). FLIM data were analyzed using OriginPro, version 8.1 (OriginLab Corporation) and FLIMfit (www.flimfit.org).

For manuscripts utilizing custom algorithms or software that are central to the research but not yet described in published literature, software must be made available to editors and reviewers. We strongly encourage code deposition in a community repository (e.g. GitHub). See the Nature Portfolio guidelines for submitting code & software for further information.

Data

Policy information about availability of data

All manuscripts must include a data availability statement. This statement should provide the following information, where applicable:

- Accession codes, unique identifiers, or web links for publicly available datasets
- A description of any restrictions on data availability
- For clinical datasets or third party data, please ensure that the statement adheres to our policy

X-ray crystallographic coordinates and data files of designed SP dimer proteins were deposited at the Protein Data Bank (PDB) with accession codes 7UNJ (SP1 with ZnPPaM bound), 7UNH (SP2, apo-state), 7UNI (SP2 with ZnPPaM bound), and 8EVM (SP3x, apo-state). All previously-published high-resolution structures referenced in this manuscript including the Blastochloris viridis RC-LH1 complex (PDB ID: 6ET5) are available at the PDB. An electron microscopy map of the full ZnPPaM-binding nanocage (Map #1) was deposited in the Electron Microscopy Data Bank with accession code EMD-40208, and a backbone model was deposited in the Protein Data Bank with accession code 8GLT. An electron microscopy map of the ZnPPaM-binding region of the nanocage (Map #2) was deposited in the Electron Microscopy Data Bank with accession code EMD-40209. Computational data related to MD simulations and circular dichroism calculations have been deposited in the ioChem-BD database (Álvarez-Moreno et al. 2015), and are accessible through the doi:10.19061/iochem-bd-6-268. Source data are provided with this paper.

Human research participants

Policy information about studies involving human research participants and Sex and Gender in Research.

Reporting on sex and gender	N/A
Population characteristics	N/A
Recruitment	N/A
Ethics oversight	N/A

Note that full information on the approval of the study protocol must also be provided in the manuscript.

Field-specific reporting

Please select the one below that is the best fit for your research. If you are not sure, read the appropriate sections before making your selection.

☒ Life sciences ☐ Behavioural & social sciences ☐ Ecological, evolutionary & environmental sciences

For a reference copy of the document with all sections, see nature.com/documents/nr-reporting-summary-flat.pdf

Life sciences study design

All studies must disclose on these points even when the disclosure is negative.

Sample size	No statistical methods were used to pre-determine sample size. The number of proteins designed and ordered for testing (48) was limited by our ability to characterize them. This sample size was found to be sufficient, as we were able to identify 3 proteins with high affinities for the target chromophore dimer.
Data exclusions	Data were not excluded from analysis.
Replication	UV/vis and CD spectra were collected at least twice on independently-prepared samples as indicated in the manuscript. All experiments described in the main text except X-ray crystallographic and cryo-electron microscopic experiments were replicated at least once. All attempts at replication were successful.
Randomization	Randomization was not relevant to this study. Group allocation was done by dividing purified protein samples and preparing control and experimental groups from the same starting material (for example, by adding a chromophore to one sample and not the other).
Blinding	Researchers were not blinded to experiments described in this work. Data sets were analyzed using objective and quantitative methods making blinding unnecessary.

Reporting for specific materials, systems and methods

We require information from authors about some types of materials, experimental systems and methods used in many studies. Here, indicate whether each material, system or method listed is relevant to your study. If you are not sure if a list item applies to your research, read the appropriate section before selecting a response.

Materials & experimental systems

n/a	Involved in the study
☒	☐ Antibodies
☒	☐ Eukaryotic cell lines
☒	☐ Palaeontology and archaeology
☒	☐ Animals and other organisms
☒	☐ Clinical data
☒	☐ Dual use research of concern

Methods

n/a	Involved in the study
☒	☐ ChIP-seq
☒	☐ Flow cytometry
☒	☐ MRI-based neuroimaging
